# Integrated Experimental and Computational Profiling of Curcumin-Derived Diarylpentanoids Reveals Mechanistic Determinants of COX1/COX2 Inhibition and Selectivity

**DOI:** 10.1021/acsomega.5c13194

**Published:** 2026-06-03

**Authors:** Mohammad Nazri Abdul Bahari, Nurul Hana Mas’od, Kamal Rullah, Mohd Fadhlizil Fasihi Mohd Aluwi, Kok Wai Lam, Faridah Abas, Wan Mardhiyana Wan Ayub, Muhamad Arif Mohamad Jamali, Asrulnizam Abd Manaf, Syahida Ahmad

**Affiliations:** † Collaborative Microelectronic Design Excellence Center, 26689 Universiti Sains Malaysia, Persiaran Bukit Jambul, Bayan Lepas, Pulau Pinang 11900, Malaysia; ‡ Faculty of Science and Technology, 65190 Universiti Sains Islam Malaysia, Nilai, Negeri Sembilan Darul Khusus 71800, Malaysia; § Kulliyyah of Pharmacy, International Islamic University Malaysia, Kuantan Campus, Jalan Sultan Ahmad Shah, Bandar Indera Mahkota, Kuantan, Pahang Darul Makmur 25200, Malaysia; ∥ Faculty of Industrial Sciences and Technology, Universiti Malaysia Pahang Al-Sultan Abdullah, Kuantan, Pahang 26600, Malaysia; ⊥ Centre for Drug and Herbal Development, Faculty of Pharmacy, Universiti Kebangsaan Malaysia, Jalan Raja Muda Abdul Aziz, Kuala Lumpur 50300, Malaysia; # Natural Medicines and Products Research Laboratory, Institute of Bioscience, Universiti Putra Malaysia, UPM Serdang, Serdang, Selangor 43400, Malaysia; ∇ Faculty of Biotechnology and Biomolecular Sciences, 37449 Universiti Putra Malaysia, UPM Serdang, Serdang, Selangor 43400, Malaysia

## Abstract

The nonsteroidal anti-inflammatory drugs (NSAIDs) reduce prostaglandin E_2_ (PGE_2_) levels by inhibiting COX2. Nevertheless, many also suppress COX1, which leads to gastrointestinal side effects. Curcumin is an anti-inflammatory agent whose bioavailability is low, necessitating the development of more active and stable curcumin analogues. In this study, 43 derivatives are evaluated for their effects on PGE_2_ production and COX regulation in IFN-γ/LPS-stimulated RAW 264.7 macrophages, and among these derivatives, three compounds (C25, C27, and C43) demonstrated strong, dose-dependent inhibition of PGE_2_, with IC_50_ values of 6.15, 5.78, and 12.15 μM, respectively outperforming curcumin and exhibiting very low cytotoxicity (IC_50_ > 500 μM). Other than that, QSAR analysis revealed that the electron-withdrawing groups, aryl substitution patterns, and higher lipophilicity contribute to enhanced PGE_2_ inhibition. Docking showed that C25 and C43 formed hydrophobic, π-cation, and halogen interactions in COX2, which are in line with their superior biological activity. The 500 ns MD simulations demonstrated that C25 and C43 stabilized COX2 more effectively than COX1, proven by the lower RMSD, reduced residue fluctuations, and stable Rg profiles. MM/PBSA also corroborated their improved affinity, with C25 having the most desirable binding free energies with both isoforms and a clear energetic preference for COX2. Altogether, the computed and biological data all indicate that C25 and C43 have the potential as COX2-selective anti-inflammatory agents, which are more potent and selective than curcumin.

## Graphical abstract

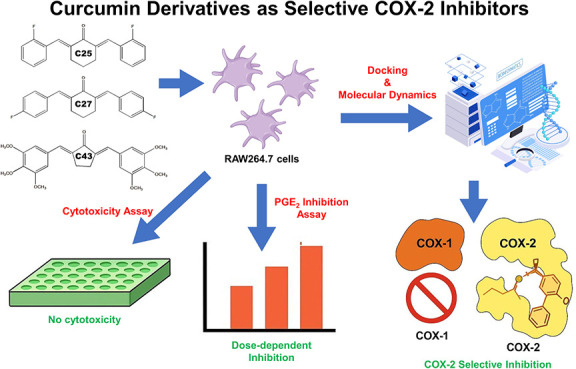

## 1 Introduction

Inflammation is a basic biological reaction that is triggered to remove detrimental stimuli and maintain the homeostasis of tissues. The bioactive lipid prostaglandin E_2_ (PGE_2_), which is an end product of arachidonic acid through the cyclooxygenase (COX) pathway, is one of the main mediators of this process.^1^ The COX enzyme family comprises two major isoforms to be mentioned: the COX1 isoform, which is constitutively expressed and crucial to maintain gastric mucosal protection, platelet aggregation, and renal functionality, and COX2. which is an inducible isoform and upregulated by inflammatory signals like lipopolysaccharide (LPS), interleukin-1β, tumor necrosis factor-α, and interferon-γ.^2^ COX2 overexpression and the subsequent rise in PGE_2_ levels play a role in the pathophysiology of many chronic diseases, such as rheumatoid arthritis, inflammatory bowel disease, neurodegenerative diseases, and most cancers.^3^

Nonsteroidal anti-inflammatory drugs (NSAIDs) decrease inflammation through their main mechanism of inhibition of COX enzyme activity. The nonselective NSAIDs, however, also inhibit both COX1 and COX2, which in turn, express high levels of gastrointestinal toxicity like bleeding and ulceration.^3^ Even though the design of COX2-selective types of inhibitors (coxibs) was developed to reduce these undesirable events, cardiovascular risks associated with them have brought up the need for safer COX2-selective molecules with better pharmacological characteristics.^4^ In turn, the development of new anti-inflammatory agents that can selectively regulate the COX2 activity is still a top research priority.

The primary polyphenol, curcumin, of *Curcuma longa*, has been documented to have anti-inflammatory, antioxidant, anticancer, antimicrobial, and neuroprotective properties.^5^ As many studies have revealed, curcumin inhibits the COX2 expression and PGE_2_ synthesis in activated macrophages.^6^ Although curcumin has potentially favorable biological outcomes, it has low bioavailability, which is not only due to low aqueous solubility but also due to a high rate of metabolism in the intestine and a high degree of hepatic conjugation, leading to minimal systemic exposure.^7^ Such constraints have motivated extensive medicinal chemistry efforts to produce curcumin analogues, which are more stable, more potent, and have improved pharmacokinetic properties.

Among such analogues are the diarylpentanoids, which have emerged as a particularly promising class. These compounds preserve the crucial pharmacophore of curcumin and enhance the chemical stability of the compound by substituting the unstable β-diketone heptanoid linker with a five-carbon spacer.^8^ Diarylpentanoids and their derivatives have also been shown to have strong anti-inflammatory effects, including the inhibition of nitric oxide, COX2, NF-κB signaling, and proinflammatory cytokines.^9^ Several studies also report superior anticancer and antioxidant effects relative to curcumin.^10^ Notably, the alterations in the aromatic rings, especially the electron-withdrawing substituents, have been demonstrated to increase the binding affinity with the COX2 and pharmacological selectivity.^11^

With these advances, the structurally optimized curcumin derivatives are a worthy reference in the creation of a new generation of COX2 inhibitors. Nevertheless, there are limited systematic studies that combine cell-based experimental methods, enzymatic COX profiling, quantitative structure–activity relationship (QSAR) modeling, molecular docking, molecular dynamics (MD), and MM/PBSA energy calculations altogether. The multilevel approach is crucial to outline the effect of structural changes on biological activity, binding energetics, and selectivity of COX isoforms.

This work involved testing 43 curcumin analogues of diarylpentanoid compounds with anti-inflammatory activities in LPS/IFN-γ-stimulated RAW 264.7 macrophages. Their impact on PGE_2_ synthesis, cytotoxicity, the enzymatic activity of COX1/COX2, and gene expression was evaluated in a systematic manner. In order to reveal the underlying molecular mechanism of such events, we also performed detailed computational studies, such as QSAR modeling, molecular docking, long-time-scale molecular dynamics, and MM/PBSA binding energy calculations of COX1 and COX2. This comprehensive experimental–computational model offers mechanistic information on the structural determinants of the COX selectivity and finds effective lead candidates to continue the development of anti-inflammatory drugs.

## 2 Materials and Methods

### 2.1 Chemical Synthesis

Synthesis of the curcumin derivative was done as described in previous research,^12^ involving the condensation of an aromatic aldehyde with three ketone types: acetone (1–18), cyclohexanone (19–33), and cyclopentanone (34–43) via a base-catalyzed aldol reaction at a 2:1 aldehyde-to-ketone ratio. Figure 1 shows a schematic representation of the synthesis. A mixture of the appropriate substituted aromatic aldehyde (20 mmol, 2 equiv) and ketone (10 mmol, 1 equiv) was dissolved in 15 mL of absolute ethanol (EtOH) and stirred at 5 °C. A 40% (w/v) aqueous sodium hydroxide (NaOH) solution was then added dropwise over several minutes, and the reaction mixture was stirred at room temperature for 10 h. The reaction was subsequently neutralized using dilute hydrochloric acid (HCl, <0.05 M), resulting in the formation of a precipitate. The solid product was collected by suction filtration, and the crude product was further purified by recrystallization from an appropriate solvent. The synthesized compounds were obtained in moderate to good yields, typically ranging from 55% to 80%, depending on the substituents and ketone scaffold.

**Figure 1:**
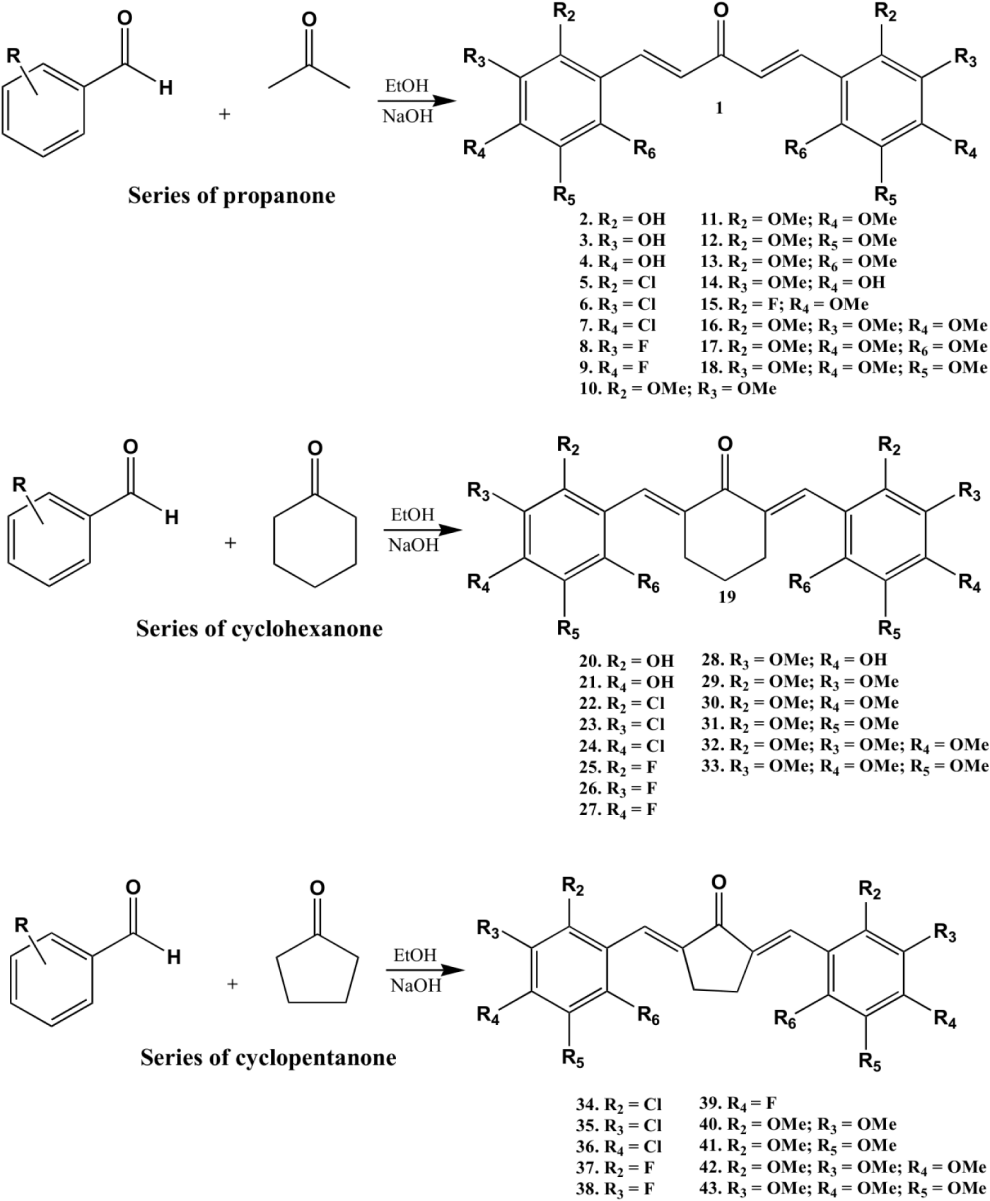
Synthesis scheme of curcumin derivatives (adapted from Chen et al., 2021 ^12^ under the Creative Commons Attribution 4.0 License (CC BY 4.0)).

### 2.2 Cell Culture, Treatment, and Assessment of PGE_2_ Production and Cell Viability

The RAW 264.7 cell line of murine macrophages was obtained from the American Type Culture Collection (ATCC, USA) and cultured in Dulbecco Modified Eagle Medium (DMEM, Sigma-Aldrich, USA) with 10% fetal bovine serum (FBS; JR Scientific Inc., USA) and 1% penicillin-streptomycin (Invitrogen Life Technologies, USA). Next, in 75 cm^2^ culture flasks (TPP, Switzerland), cells were cultured at 37 °C in a humidified incubator with 5% CO_2_ (ESCO, Singapore). When the confluency was 80–90%, cells were trypsinized and seeded in 96-well plates (Jet Biofil, China) at a concentration of 5 × 10^4^ cells/well and left to incubate overnight. The macrophages were then incubated with 5 μg/mL lipopolysaccharide (LPS; *Escherichia coli* serotype 055, Sigma-Aldrich) and 100 μg/mL recombinant mouse interferon-γ (IFN-γ; Sigma-Aldrich, USA), then treated with the test compounds (0.78–200 μM) or vehicle control for 17 h. NS-398 was included as an anti-inflammatory positive control. After incubation, the cell culture supernatants were collected for the determination of prostaglandin E_2_ (PGE_2_) levels using the Prostaglandin E_2_ Express ELISA Kit (Cayman Chemical, Ann Arbor, USA) according to the manufacturer’s instructions. The percentage of PGE_2_ inhibition was calculated by using the following equation:

$$\%{\mathrm{PGE}}_{2}\;\mathrm{inhibition}=\frac{{[{\mathrm{PGE}}_{2}]}_{\mathrm{control}}-{[{\mathrm{PGE}}_{2}]}_{\mathrm{sample}}}{{[{\mathrm{PGE}}_{2}]}_{\mathrm{control}}}\times 100$$

After PGE_2_ quantification, cell viability was determined by applying the MTT assay. 100 μL of DMEM and 20 μL of 5 mg/mL MTT solution were added to each well and incubated for 3 h to allow the formation of purple formazan crystals. Subsequently, the culture medium was discarded and 100 μL of 100% dimethyl sulfoxide (DMSO) was added to solubilize the dye. A microplate reader was used to obtain the absorbance at 570 nm. The percentage of cell viability was determined as follows:

$$\%\mathrm{cell viability}=\frac{{[\mathrm{OD}]}_{\mathrm{sample}}}{{[\mathrm{OD}]}_{\mathrm{control}}}\times 100$$

The 50% cytotoxic concentration (CC_50_) was defined as the concentration of the compound that reduced cell viability to 50% relative to untreated control cells. CC_50_ values were calculated using nonlinear regression analysis of dose–response curves generated from MTT absorbance measurements.

### 2.3 COX Enzymatic Inhibition by Curcumin Derivatives

The inhibitory activity of curcumin derivatives against COX enzymes was evaluated using the COX Inhibitor Screening Assay Kit (Cayman Chemical, USA), according to the manufacturer’s instructions. The workflow was implemented in a plate-based format, and the COX activity was quantified by measuring PGE_2_ generated in the reaction using the same PGE_2_ immunoassay readout described in Section 2.2. Apart from this plate-based handling and downstream PGE_2_ quantification step, all of the key assay conditions followed the kit protocol. Fourteen compounds showing the highest PGE_2_ inhibition were screened at 100 μM. Compounds with >50% inhibition were further tested at 3.13–25 μM (2-fold dilutions), while those with <50% inhibition were tested at 12.5–100 μM. Aspirin (50 μM) was used as a positive control for COX1 enzymatic inhibition, while NS-398 (2 μM) was used as the selective COX2 control. The selected aspirin concentration reflects its established inhibitory range in enzymatic assays.^13^ PGE_2_ levels were quantified as described in Section 2.2, while COX inhibition (%) was calculated based on PGE_2_ concentration using the following formula:

$$=\frac{{[{\mathrm{PGE}}_{2}]}_{\mathrm{COX}100\%\mathrm{activity}}-{[{\mathrm{PGE}}_{2}]}_{\mathrm{sample}}}{{[{\mathrm{PGE}}_{2}]}_{\mathrm{COX}100\%\mathrm{activity}}}\times 100$$

### 2.4 RT-qPCR Analysis of COX1 and COX2 Expression

Quantitative real-time PCR (qRT-PCR) was used to evaluate the effect of compounds 25, 27, and 43 on the gene expression of COX1 and COX2 at concentrations of 2, 10, and 50 μM in RAW264.7 cells. COX1 and COX2 stimulation was done using 10 nM PMA or 100 U/mL IFN-γ and 5 μg/mL, respectively. Total RNA was extracted from treated cells using the RNeasy Plus Mini Kit (Qiagen, Germany), and its purity and concentration were then measured using a NanoDrop Lite Spectrophotometer (Thermo Scientific, USA) and verified by gel electrophoresis. After that, 1 μg of RNA was reverse-transcribed with the iScript Reverse Transcription Supermix (Bio-Rad, USA), and qRT-PCR was then conducted using the iTaq Universal SYBR Green Supermix (Bio-Rad, USA) in 20 μL reactions containing 200 nM primers and 50 ng of cDNA. Table S1 gives the primer sequences of COX1 and COX2. Amplification was done on a Bio-Rad CFX96 system under the following conditions: 95 °C for 3 min, followed by 40 cycles of 95 °C for 10 s, and gene-specific annealing for 30 s. A dissociation (melt) curve analysis was performed at the end of each run to confirm amplification specificity and the absence of primer–dimer artifacts. Expression levels were normalized to the internal reference genes GAPDH and β-actin, which were selected as housekeeping controls to reduce normalization bias and improve robustness under inflammatory stimulation conditions. Each sample was analyzed in technical triplicate, and the entire experiment was independently repeated three times (*n* = 3).

### 2.5 Statistical Analysis

All data are presented as mean ± SEM from at least three independent experiments (*n* = 3), unless otherwise stated. Statistical analyses were performed using one-way analysis of variance (ANOVA), followed by Tukey’s multiple comparison post hoc test to determine differences between groups. A *P* value of less than 0.05 was considered to be statistically significant. IC_50_ and CC_50_ values were calculated by nonlinear regression analysis of dose–response curves using GraphPad Prism

### 2.6 Quantitative Structure–Activity Relationship

Curcumin derivatives were first separated into a training set (19 compounds) and a test set (8 compounds). The IC_50_ values (μM) were expressed in molar units and converted to negative logarithm (−log IC_50_ or pIC_50_) for QSAR analysis. ChemDraw Pro v8.0 (Cambridge, USA) was used to draw the molecular structures. Energy minimization was performed using the CHARMM force field until the root-mean-square gradient value was less than 0.001 kcal/mol·Å. Using Discovery Studio 3.1 (Accelrys, USA), the optimized structures were used to calculate physicochemical descriptors, including structural, thermodynamic, steric, electronic, and quantum mechanical properties. Highly correlated descriptors were excluded.

Genetic Function Approximation (GFA) generated 100 regression models through 10000 crossovers, which linked biological activity to molecular descriptors. Using Friedman’s lack-of-fit (LOF), *R* ^2^, adjusted *R* ^2^, predicted *R* ^2^, and *Q* ^2^, models were evaluated and the best model with ≤5 terms was selected based on the lowest LOF and highest predictive power. External validation followed Roy (2007):

$$R_{\mathrm{Pred}}^{2}=1-\frac{\sum {\left(Y_{\mathrm{Pred}\left(\mathrm{test}\right)}-Y_{\mathrm{Pred}\left(\mathrm{test}\right)}\right)}^{2}}{\sum {\left(Y_{\mathrm{Pred}\left(\mathrm{test}\right)}-{\mathrm{Y̅}}_{\mathrm{Training}}\right)}^{2}}\times 100$$

where *Y*_Pred(test)_ and *Y*_Obs(test)_ are the predicted and observed activity values of test set, respectively, whereas *Y̅*_Training_ is the mean activity value of the training set.

### 2.7 Molecular Docking and Molecular Dynamics Simulations

First, the crystal structures of COX1 (PDB ID: 6Y3C) and COX2 (PDB ID: 5F1A) were originally retrieved from the Protein Data Bank, and the PDBs were then prepared by retaining the heme prosthetic group essential for catalysis, while crystallographic waters, buffer ions, glycosylation residues, and other nonessential heteroatoms were removed to avoid artifacts during conformational sampling.^14^ Curcumin derivatives, compound 43 (PubChem CID: 2308087), compound 27 (CID: 1551384), and compound 25 (CID: 20416677), were downloaded in SDF format and converted to PDB using OpenBabel.^15^ Molecular docking was performed using AutoDock Vina v1.2.4., which predicts ligand binding poses and affinities by optimizing a hybrid scoring function through an efficient gradient-based conformational search.^16^ From the docking results, the top models that possess good interaction geometry and docking scores were selected as the starting structures for MD simulations.

Next, the models were introduced to a solvated system, which was carried out in CHARMM-GUI^17^ using the CHARMM36m all-atom force field^18^ and TIP3P explicit solvent.^19^ Here, each complex was solvated in a rectangular box with a minimum of 10 Å water buffer and neutralized with 0.15 M NaCl. All MD simulations were then conducted on each model using GROMACS 2025.2.^20^ For energy minimization, which is prior to MD simulations, the steepest-descent algorithm was used (5,000 steps; convergence criterion = 1,000 kJ·mol^–1^·nm^–1^), with the backbone and side-chain atoms restrained with force constants of 400 and 40 kJ·mol^–1^ ·nm^–2^, respectively. Particle Mesh Ewald (PME) with a cutoff of 1.2 nm was used to treat long-range electrostatics, while van der Waals forces were modeled with a force-switching function between 1.0 and 1.2 nm. LINCS was used to constrain all the covalent bonds that included hydrogens. After that, equilibration was performed using restrained NVT dynamics for 125 ps at 303.15 K (V-rescale thermostat), which was then followed by NPT equilibration at 1 bar using the C-rescale barostat (τ_p_ = 5.0 ps; compressibility = 4.5 × 10^–5^ bar^–1^). A simulation of production was then run at 500 ns on a 2 fs time step under the md integrator. The temperature and pressure were kept at 303.15 K and 1 bar, respectively. Verlet cutoff scheme was implemented with neighbor lists being updated after every 20 steps. Trajectory coordinates were recorded at 100 ps intervals, and lower frequency energy recording was used to save on computation time. All trajectory analyses specifically the root-mean-square deviation (RMSD), root-mean-square fluctuation (RMSF), radius of gyration (*R*_g_), and protein–ligand hydrogen bonding were all performed using standard GROMACS tools. Finally, the VMD^21^ and UCSF Chimera^22^ were used for visual inspection and interaction analysis.^23,25^

### 2.8 MM/PBSA

The molecular mechanics/Poisson–Boltzmann surface area (MM/PBSA) framework implemented in gmxMMPBSA23, which is fully compatible with GROMACS trajectories and the CHARMM36m force field, was used to compute the binding free energies for each COX–ligand complex. For this, 5,000 snapshots were sampled to provide a statistically significant sampling of the conformational space in the final 500 ns production phase at intervals of 5 frames. Calculations were done using the standard single-trajectory protocol, whereby complex, ligand, and protein states are calculated based on the same aligned trajectory to reduce structural noise. The Poisson–Boltzmann (PB) model was used to calculate polar solvation energies at an ionic strength of 0.15 M, a grid fill ratio of 4.0, and CHARMM-optimized atomic radii (PBRadii = 7, radiopt = 0). SASA was used to determine the contributions of nonpolar processes by the default surface tension parameters. The total binding energies were calculated as the summation of van der Waals, electrostatic, polar solvation, and nonpolar solvation.

## 3 Results

### 3.1 Effects of Curcumin Derivatives on COX Activity, PGE_2_ Production, and Cell Viability

The production of PGE_2_ was significantly higher in the case of stimulation of RAW264.7 macrophages with IFN-γ/LPS compared to the case of unstimulated cells (21.34 ± 17.22 pg/mL vs 1049.39 ± 120.45 pg/mL). A total of 43 curcumin derivatives were screened, and 10 compounds were found to effectively inhibit the formation of PGE_2_ in a dose-dependent manner, with compound 25 being the most potent (Figure 2). Increasing concentrations of compound 25 (0.78–50 μM) reduced PGE_2_ levels from 1009.28 ± 116.33 to 36.40 ± 16.24 pg/mL (Figure 2A).

**Figure 2:**
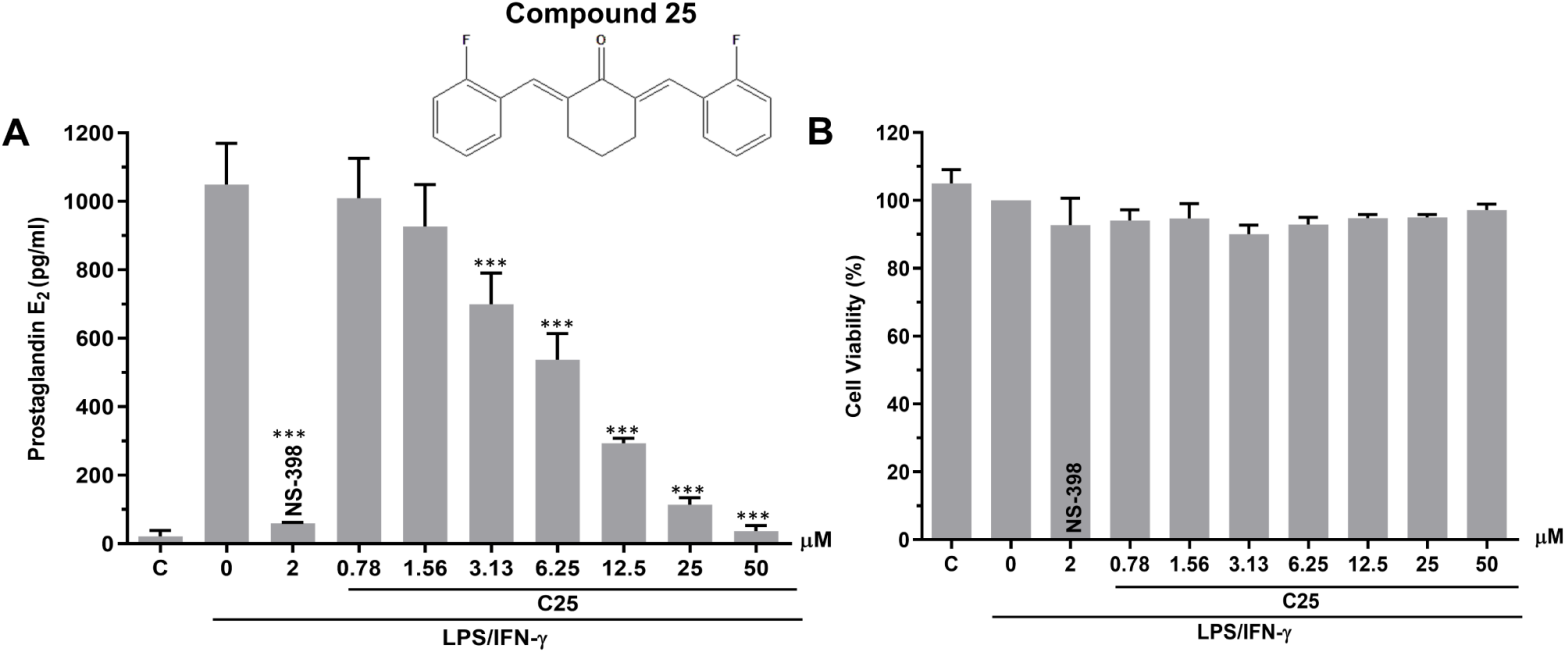
Effects of compound 25 on PGE_2_ production and cell viability in IFN-γ/LPS-stimulated RAW264.7 cells. RAW264.7 cells were incubated with recombinant murine IFN-γ (100 U/mL) and LPS (5 μg/mL), then treated with different concentrations of compound 25 (24 h). NS-398 (2 μM) represents a positive control. “C” represents the basal (unstimulated) control group, while “0” represents IFN-γ/LPS-stimulated cells without compound treatment. (A) PGE_2_ levels in the culture supernatant were measured using an enzyme immunoassay (EIA). (B) Cell viability was determined under the same treatment conditions. The values are presented as mean ± SEM from three independent experiments (*n* = 3). ***P < 0.001 indicates a significant difference compared to the IFN-γ/LPS-stimulated control group (“0”).

The determination of the IC_50_ revealed that ten compounds had a strong PGE_2_ inhibition (IC_50_ = 5.17–14.39 μM), which is much better than curcumin (IC_50_ = 18.23 ± 2.25 μM), while NS-398 demonstrated the expected high potency (IC_50_ = 0.11 ± 0.05 μM). The evaluation of cytotoxicity showed that the majority of the derivatives were nontoxic at the concentrations tested, with CC_50_ values exceeding 50 μM. Compound 25 maintained cell viability above 80% across the tested concentration range (Figure 2B). Similarly, other active derivatives exhibited CC_50_ values exceeding 50 μM (Table 1), indicating low cytotoxicity under the tested conditions.

**Table 1:**
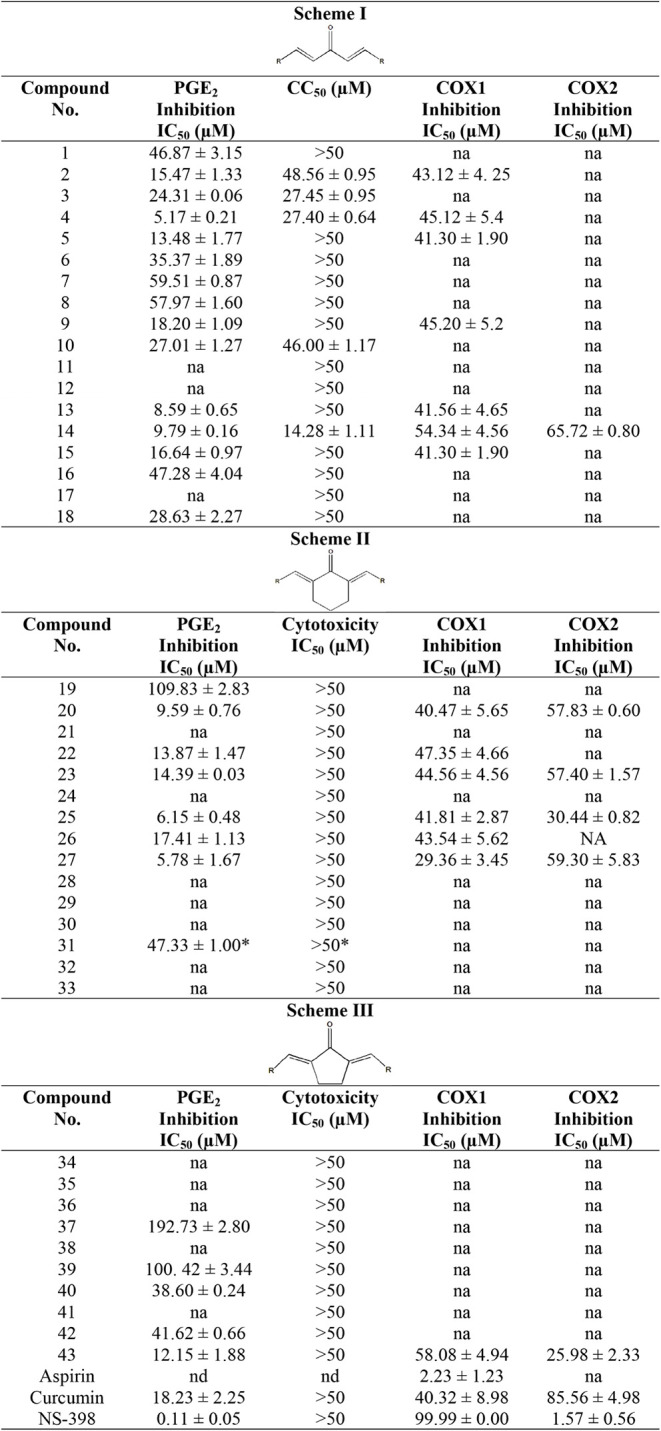
IC_50_ Values of PGE_2_ Inhibition, Cytotoxicity, and COX Activity of Curcumin Derivatives^a^

a Aspirin was a positive control for the COX1 enzymatic inhibition assay. Aspirin IC_50_ value was determined from dose–response curves in the enzymatic inhibition assay. Cytotoxicity of aspirin was not determined as it was used solely as a reference inhibitor in the enzymatic inhibition assay. NS398 was used as positive control for PGE_2_ inhibition and COX2 enzymatic inhibition assay. Curcumin was used as the reference compound. na: not active. nd: not determined.

In order to find out whether PGE_2_ suppression was associated with direct inhibition of COX enzymatic activity, derivatives with IC_50_ <20 μM in the PGE_2_ assay were evaluated for COX1 and COX2 inhibition. The majority of the compounds selectively inhibited COX1 at 100 μM (65.32–81.14% inhibition), while the COX2 inhibition was more diverse (31.3–87.27%). Compound 27 was found to be selective for COX1 (IC_50_: 29.36 ± 3.45 μM for COX1; 59.30 ± 5.83 μM for COX2). Meanwhile, compounds 25 and 43 exhibited greater selectivity for COX2 (Table 1). Among all, compound 43 was the most preferred, since it possessed the lowest IC_50_ for COX2 (25.98 ± 2.33 μM) versus COX1 (58.08 ± 4.94 μM). These activities were compared with aspirin (COX1 control: IC_50_ = 2.23 ± 1.23 μM) and NS-398 (COX2 control: IC_50_ = 1.57 ± 0.56 μM), with curcumin exhibiting moderate inhibition of both the isoforms.

### 3.2 Quantitative Structure–Activity Relationship (QSAR) Analysis

The QSAR modeling was done to determine the structural determinants regulating PGE_2_ inhibition within the curcumin derivatives. A Genetic Function Approximation model (eq 1) was generated using 19 training compounds and validated using 8 test compounds. It was shown that the model exhibited high internal consistency (adjusted *R* ^2^ = 0.661) and good external predictivity (*Q* ^2^ = 0.883), which means that the descriptors selected in this model are reliable predictors of biological activity (Figure 3). The observed activity was in close agreement with predicted pIC_50_ values (RMS residual error = 0.142), in which the small difference between Adjusted *R* ^2^ and Predicted *R* ^2^ (<0.3) suggests minimal overfitting (Table 2). The Friedman LOF of 0.240 further indicates the robustness of the model.

**Figure 3:**
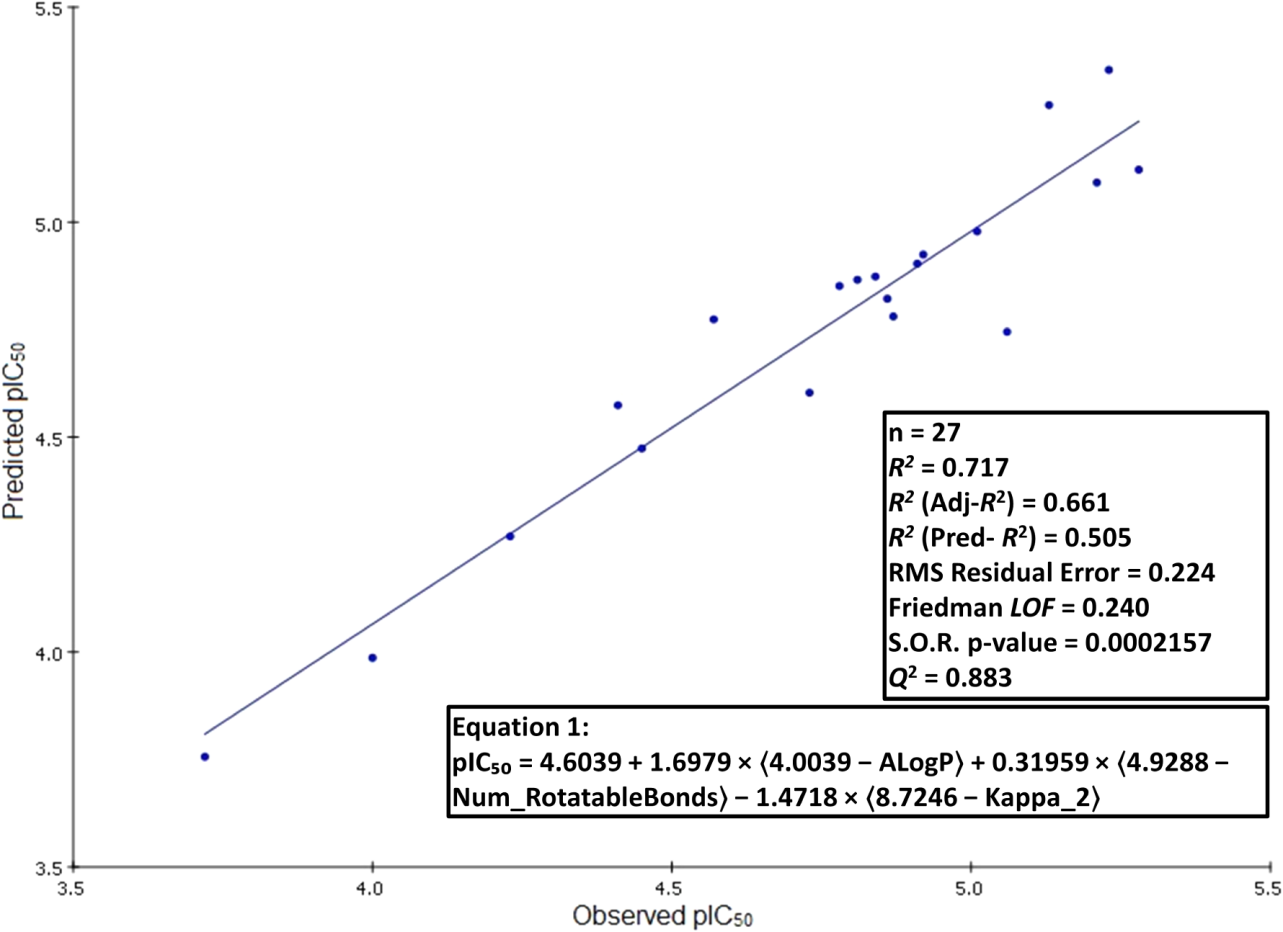
Graph of predicted pIC_50_ values against observed pIC_50_ values for the model developed by eq 1.

**Table 2:** Observed and Predicted pIC_50_ of Curcumin Derivatives from Three Models Obtained from the GFA Method

Compound No.	Observed pIC_50_	Predicted pIC_50_	Residual (±)
*Training set*			
1	4.33	4.168	0.162
2	4.81	5.045	0.235
4	5.28	5.045	0.235
5	4.87	4.488	0.382
6	4.45	4.488	0.038
7	4.23	4.488	0.258
8	4.24	4.488	0.248
15	4.78	4.604	0.176
18	4.54	4.604	0.064
20	5.01	5.051	0.041
21	5.13	5.051	0.079
22	4.86	5.051	0.191
24	4.92	5.051	0.131
25	5.21	5.051	0.159
27	5.23	5.051	0.179
39	4.00	4.052	0.052
40	4.41	4.604	0.194
42	4.38	4.604	0.224
43	4.91	4.604	0.306
*Test set*			
3	4.61	5.045	0.435
10	4.57	4.604	0.034
14	5.00	5.217	0.217
16	4.32	4.604	0.284
19	3.96	4.506	0.546
23	4.84	4.254	0.586
26	4.76	5.051	0.291
37	3.72	4.052	0.332

The main factors that contributed to PGE_2_ inhibition were the following three descriptors: ALogP, Num_RotatableBonds, and Kappa_2 (Table S2). Num_RotatableBonds (molecular flexibility) and ALogP (lipophilicity) were both positively correlated with potency, which is consistent with enhanced membrane permeability and ligand adaptability within the COX active site. Kappa_2 (a shape/topology parameter), in turn, had a negative correlation with inhibition, indicating that smaller or less branched structures could be more likely to bind. All predicted activity was higher in compounds that contained electron-withdrawing substituents like fluorine, especially in para or ortho positions, which is consistent with experimental trends.

### 3.3 Binding Orientation of Curcumin Derivatives in the COX Active Site

Figure 4 depicts the binding orientations of the compounds C25, C27, and C43 in the COX1 and COX2 binding pockets. All three derivatives in COX2 occupied an equivalent binding pocket (although not at the meloxicam binding site) and had rapidly overlapping interaction patterns that involved a shared set of hydrophobic and aromatic residues that form the active site, which is in line with a conserved binding mode (Figure 4b). On the contrary, the heterogeneity of binding in the COX1 pocket was lower (Figure 4a) and showed fewer common interacting residues among the derivatives (Figure S1 and Table S3a). Importantly, C43 exhibited more similarity to curcumin in the COX1 interaction profile than C25 and C27, which interacted with more distinct and dispersed sets of residues. Tables 3 and 4 present the summary of interaction counts and docking energies, and Table S3a and Table S3b provide detailed ligand–residue interaction distances between the two COX isoforms.

**Figure 4:**
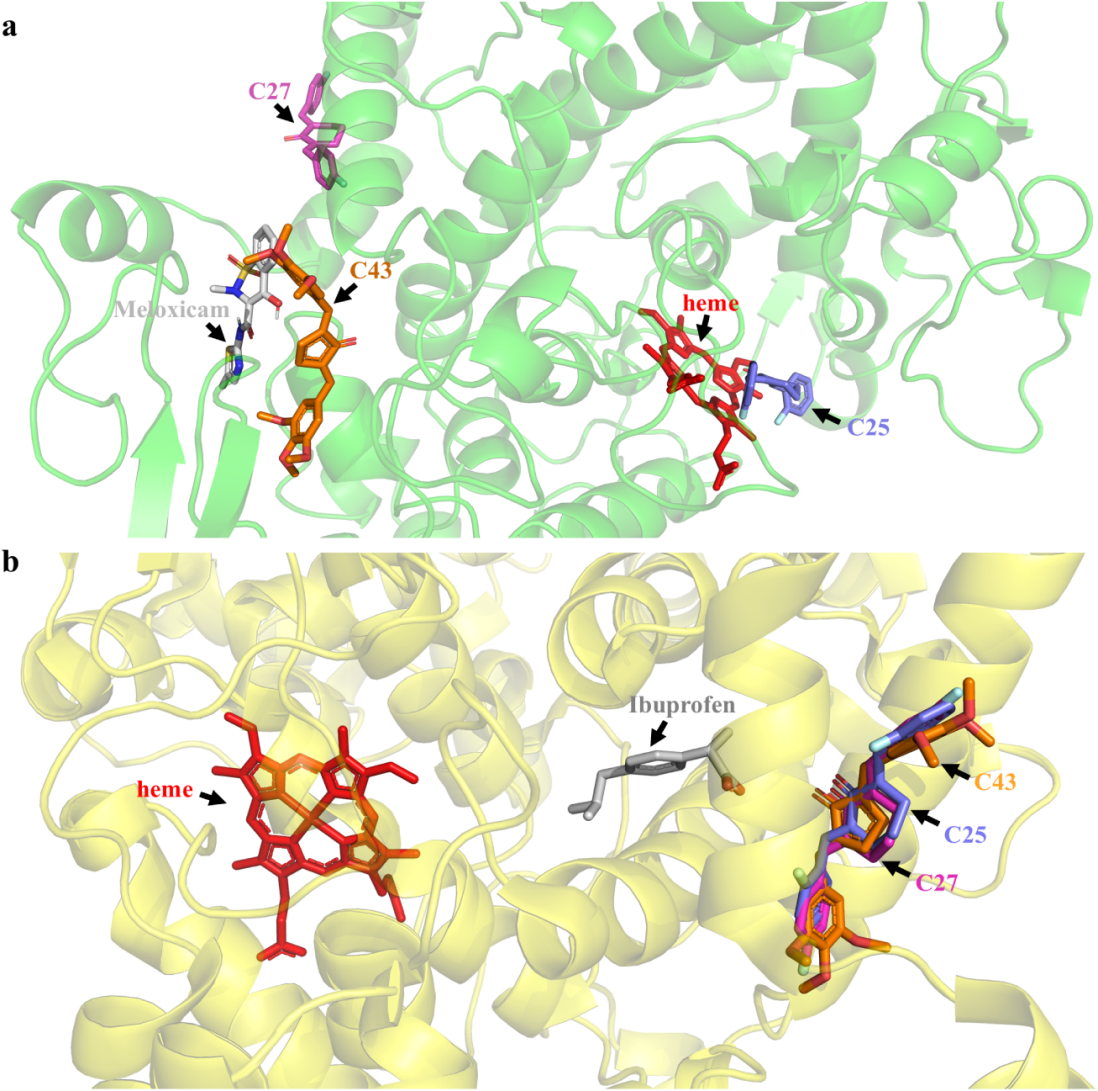
Docking poses of compounds C25 (blue), C27 (pink), and C43 (orange) within cyclooxygenase isoforms. (a) COX1 (green ribbon) with a heme group (red) and reference inhibitor meloxicam, showing heterogeneous binding with fewer shared residues, where C43 resembled curcumin more closely than C25 or C27. (b) COX2 (yellow ribbon) with a heme group (red) and reference inhibitor Ibuprofen, where all three compounds occupied an equivalent pocket and displayed overlapping interactions with conserved hydrophobic and aromatic residues.

**Table 3:** Summary of Molecular Docking of COX1 by Compounds 25, 27, and 43

	Number of Interactions						
COX1 Sample	Hydrophobic Interaction	π-Stacking	π-Cation	H-bond	Halogen Bond	Total Number of Interactions	Docking Energy (kcal/mol)
C25	6	-	-	-	-	6	–9.115
C27	9	1	-	-	-	10	–8.895
C43	6	-	-	3	-	9	–9.145
Curcumin	1	-	-	5	-	6	–10.125
Meloxicam	2	-	-	5	-	7	ND

**Table 4:** Summary of Molecular Docking of COX2 by Compounds 25, 27, and 43

COX2	Number of Interactions						
Sample	Hydrophobic Interaction	π-Stacking	π-Cation	H-Bond	Halogen Bond	Total Number of Interactions	Docking Energy (kcal/mol)
C25	5	1	1	-	1	8	–9.482
C27	5	-	1	-	-	6	–8.99
C43	4	-	-	1	1	6	–10.174
Curcumin	2	1	-	2	-	5	–10.083
Ibuprofen	5	-	-	-	-	5	ND

C27 had the most interactions in COX1 (*n* = 10) that were primarily due to a large hydrophobic interface (nine residues) and a π-stacking interaction with Trp69. C25 and C43 formed six and nine interactions, respectively. It was observed that C43 was the only residue that possessed hydrogen bonding to Arg438, Arg30, and Gln13, while C25 and C27 never adopted similar polar contacts with these residues. Curcumin (Cur), with five hydrogen bonds and a single hydrophobic contact, showed a different pattern of interaction, and this aligns with its more polar scaffold. Even though there were variations in interaction profiles, the docking energies of the derivatives were comparable (−8.895 to −9.145 kcal/mol), but curcumin scored slightly more favorably at −10.125 kcal/mol. These results suggest that the derivatives rely more on hydrophobic anchoring than on polar contacts for COX1 binding.

There was strong and stable binding in all of the derivatives within COX2. C25 had a total of eight interactions, including hydrophobic, π-stacking, π-cation, and one halogen bond, with a docking score of −9.482 kcal/mol. C27 had six interactions, with the preeminent one being hydrophobic, with a docking score of −8.990 kcal/mol. In the meantime, C43 produced one hydrogen bond and one halogen bond, with a high docking energy of −10.174 kcal/mol and is the most favorable of the derivatives, but it is slightly stronger than curcumin (−10.083 kcal/mol). The reference NSAID ibuprofen interacted through five hydrophobic contacts without additional aromatic or polar interactions, which is in line with its known binding characteristics.

Overall, C25 and C43 showed more numerous and distinct patterns of interaction in COX2 than in COX1, and C27 was also found to have a similar number of interactions in both isoforms. Most importantly, a halogen bond was only observed with C25 in COX2, another stabilization interaction present in the COX2 binding pocket, whereas C43 is majorly stabilized by hydrophobic and hydrogen bonding interactions. Tight engagement of the π-cation interaction between C25 and C27 is further aided by the presence of π-cation interactions with Arg89, which is one of the important anchoring residues for the binding of NSAIDs.

### 3.4 Molecular Dynamics

To further examine the dynamic response of curcumin derivatives in the COX active sites, 500 ns all-atom MD simulations were done on both the COX1 and the COX2 complex with compounds C25, C27, and C43. Here, curcumin was considered as the reference, while the enzyme’s respective crystallographic ligands: meloxicam (MXM) with COX1 and ibuprofen with COX2 as controls. RMSD, RMSF, radius of gyration (*R*_g_), and hydrogen-bond analyses were conducted to evaluate the stability of the complexes, their residue flexibility, and interaction persistence (Figures 5 and 6).

**Figure 5:**
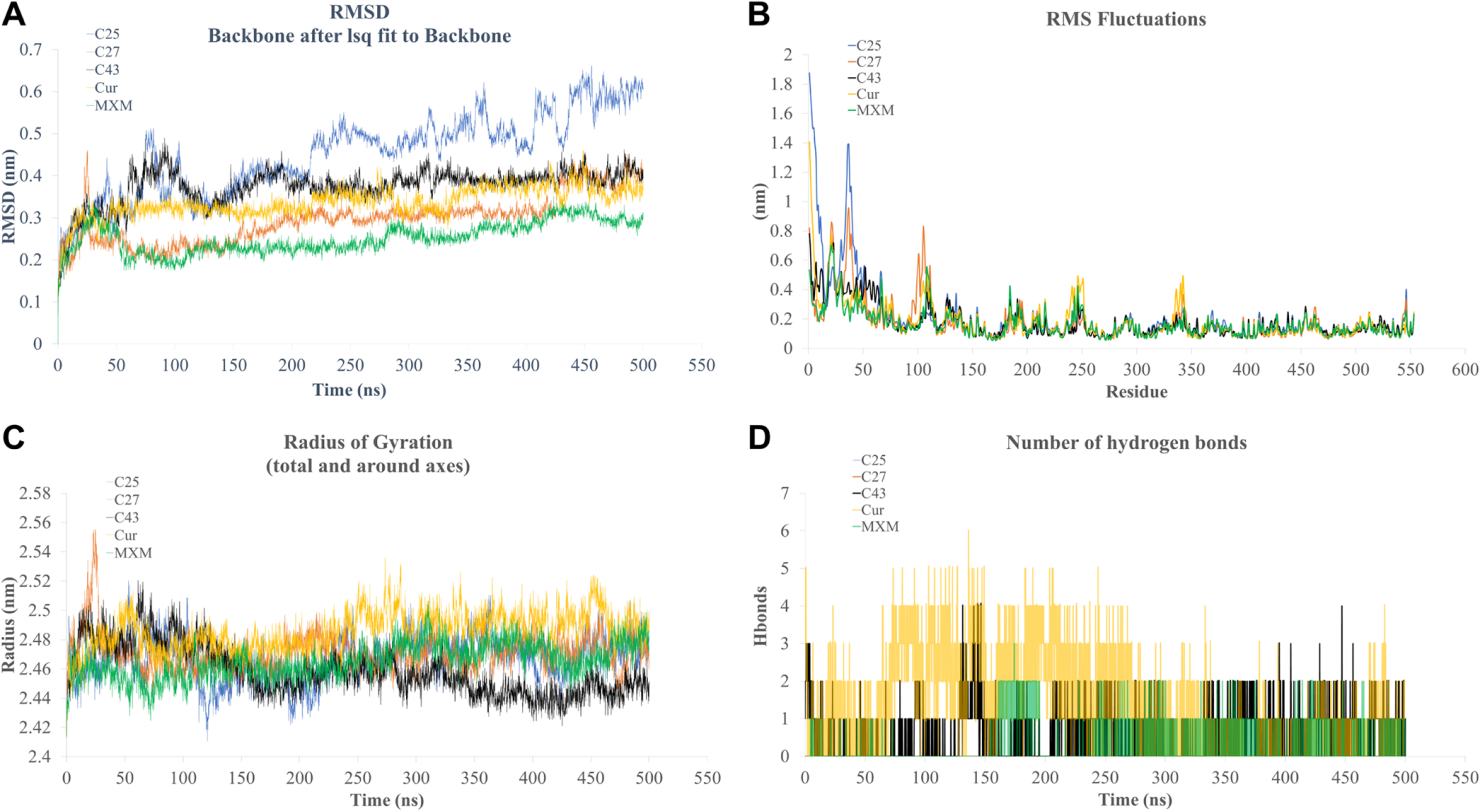
Structural dynamics of COX1 complexes with curcumin derivatives, compounds 25, 27, and 43, over 500 ns MD simulations. (a) Root mean square deviation (RMSD), (b) root mean square fluctuation (RMSF), (c) radius of gyration (*R*_g_), and (d) number of protein–ligand hydrogen bonds. Meloxicam (MXM) as the native ligand in the enzyme crystal was used as the positive control, while curcumin (Cur) was used as the reference compound.

**Figure 6:**
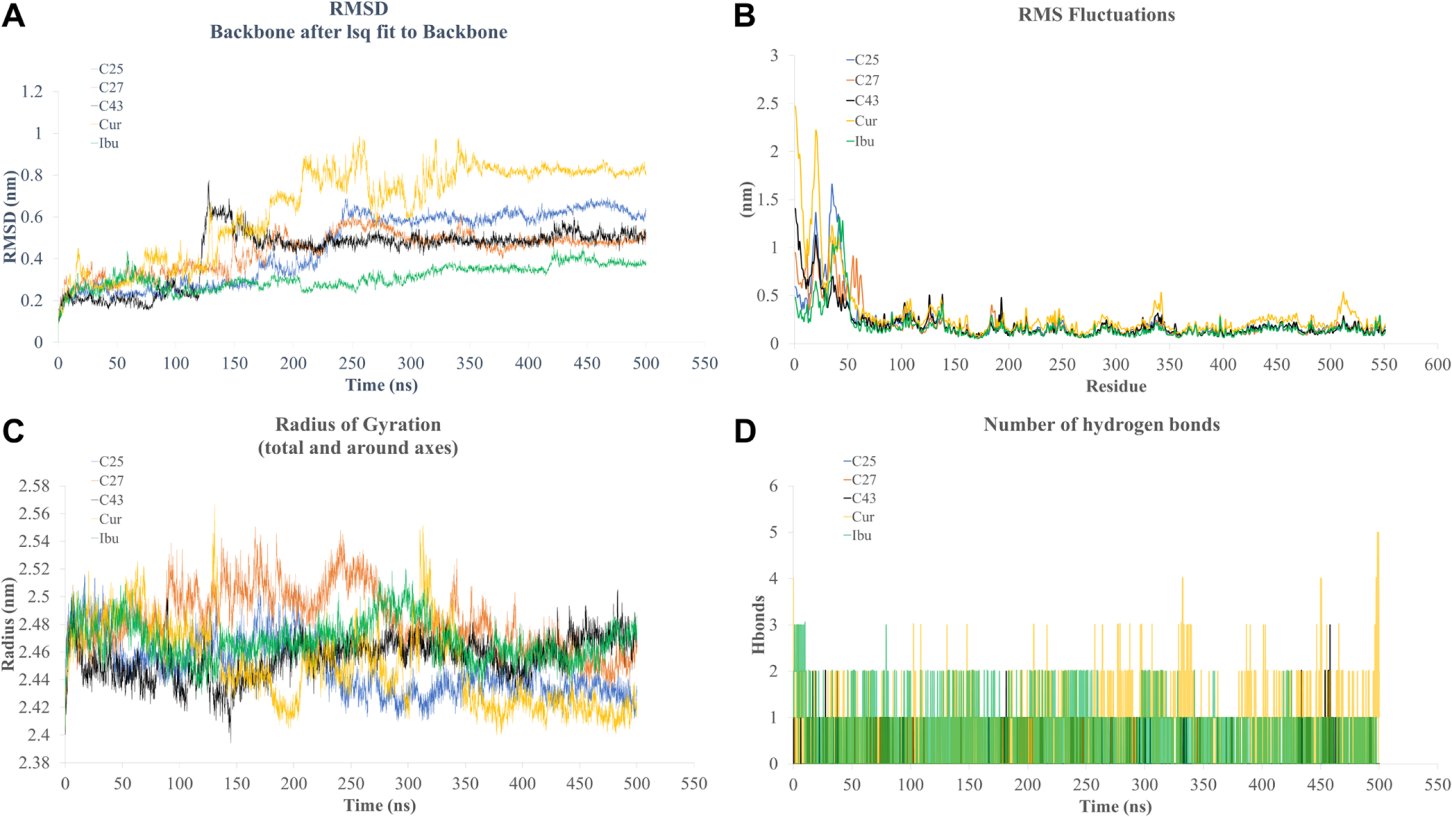
Structural dynamics of COX2 complexes with curcumin derivatives, compounds 25, 27, and 43, over 500 ns MD simulations. (a) Root mean square deviation (RMSD), (b) root mean square fluctuation (RMSF), (c) radius of gyration (*R*_g_), (d) number of protein–ligand hydrogen bonds. Ibuprofen (Ibu) as the native ligand in the enzyme crystal was used as the positive control, while curcumin (Cur) was used as the reference compound.

It was observed that all ligand–protein complexes remained structurally stable within the 500 ns time frame and maintained RMSD values below 0.5 nm; however, except for C25 in COX1, its RMSD value fluctuated between 0.40 and 0.65 nm from 220 to 500 ns. MXM possessed the lowest RMSD in COX1, implying that the protein backbone is well stabilized. This is followed by C27 and curcumin, while C43 displayed moderate fluctuations. Meanwhile, in COX2, ibuprofen and C43 displayed consistently lower RMSD between 150 and 500 ns compared to C25 and C27, which showed a slight drift after 200 ns (∼0.27 nm) but maintained stable fluctuations until 500 ns, suggesting tighter and more stable engagement within the COX2 active site. A comparatively significant drift was observed with curcumin, which concludes the more stable anchoring of the synthesized derivatives relative to the natural parental structures.

COX1 and COX2 RMSF profiles exhibited comparable trends, with expected peaks being present in loop regions and termini. The residues in the catalytic channel also had small fluctuations (<1.0 nm) when all the derivatives interacted, reflecting that there was a slight disruption to the structure of the binding site. C25 and C43 were found to have stronger stabilization of the binding pocket, which is concluded from the fact that C25 and C43 consistently restricted local residue mobility more effectively than C27 in both isoforms, consistent with their biological potency. Curcumin showed slightly elevated fluctuations observed in both COX1 and COX2, and this correlates with its weaker dynamic stabilization compared to the derivatives and positive controls.

The *R*_g_ values were between ∼2.42 and 2.54 nm, suggesting that all systems remained compact throughout the simulations. C43 retained the highest structural packing in COX1, but MXM showed mild expansion at a later time point. Complexes with C25, C43, and curcumin in COX2 had more stable *R*_g_ profiles, which suggests that the global protein architecture becomes more stabilized. The slightly higher fluctuations in *R*_g_ for ibuprofen-bound COX2 emphasize the stronger compactness-inducing effect of the synthesized derivatives, particularly C25 and C43.

The binding interaction was further distinguished by hydrogen-bond analysis, and it was found that C43 and C27 had a higher number of hydrogen bonds (an average of 2–6 bonds) in COX1 relative to the positive control, meloxicam (MXM). However, C25 and C27 showed fewer or no hydrogen bonds during the simulation. Ibuprofen and curcumin, which were used as positive controls in COX2, showed a relatively stronger formation of hydrogen bonds as compared to the synthesized derivatives. These observations indicate that hydrogen-bond interactions do not seem to be the major factor in determining the binding affinity of C25 and C27 to both COX isoforms; rather, this may be mediated by hydrophobic contacts and shape complementarity in the active site.

All of the MD analyses show that the synthesized curcumin derivatives, specifically C25 and C43, have stronger stabilizing effects on COX2 compared to those on COX1. They have lower relative RMSD, RMSF, and *R*_g_ values in critical catalytic regions, which is reflected. These derivatives created stable complexes with the enzyme that were similar to, or in certain cases more stable than, those of curcumin and the positive controls, indicating their positive dynamic behavior toward COX 2.

### 3.5 MM/PBSA

The relative affinity and binding energy of the curcumin derivatives for COX1 and COX2 were calculated using the MM/PBSA approach (Table 5). For COX1, the ligands were found to be negatively bound with prevailing van der Waals interactions, which is in line with the insertion of the aromatic scaffolds into the hydrophobic cyclooxygenase channel. C25 possessed the most negative Δ*G*_bind_ (−38.66 ± 4.84 kcal·mol^–1^) out of all derivatives, being comparable to or slightly stronger than the native inhibitor meloxicam (−30.61 ± 5.03 kcal·mol^–1^). C43 and C27 showed moderate yet stable affinities (−26.07 ± 6.85 and −25.11 ± 5.18 kcal·mol^–1^, respectively), while curcumin showed a slightly more favorable mean Δ*G*_bind_ than C27 and C43 (−27.19 ± 9.08 kcal·mol^–1^). However, there was a noticeably larger standard deviation, which indicates a more heterogeneous and dynamically labile binding mode. Even though the gas-phase vdW term was strongly stabilizing (∼−29 to −44 kcal·mol^–1^) in all COX1 complexes, the polar solvation (EPB) opposed binding for curcumin and C43, which were subjected to the largest PB penalties. Figures S2–S11 depict the MM/PBSA per-residue energy decomposition for each simulation, showing the energetic contributions of individual residues.

**Table 5:** MM/PBSA Binding Free Energy and Energy Components of Curcumin Derivatives–COX Complexes^a^ ^b^

Samples	VDWAALS	EEL	EPB	ENPOLAR	GGAS	GSOLV	TOTAL, ΔG_binding_
Cyclooxygenase-1 complexes energy components (kcal/mol)							
C25	–43.66 ± 3.67	–0.4 ± 3.69	9.33 ± 4.12	–3.94 ± 0.11	–44.05 ± 4.03	5.39 ± 4.09	–38.66 ± 4.84
C27	–29.40 ± 2.78	–2.72 ± 1.82	10.89 ± 4.59	–3.88 ± 0.17	–32.12 ± 3.56	7.01 ± 4.53	–25.11 ± 5.18
C43	–39.47 ± 4.86	–16.24 ± 9.91	34.86 ± 9.57	–5.21 ± 0.49	–55.71 ± 11.59	29.64 ± 9.49	–26.07 ± 6.85
Curcumin	–38.65 ± 9.12	–19.66 ± 11.25	35.76 ± 12.42	–4.63 ± 0.55	–58.32 ± 0.55	31.13 ± 12.03	–27.19 ± 9.08
Meloxicam*	–44.67 ± 2.64	–0.47 ± 5.53	18.37 ± 3.9	–3.85 ± 0.13	–45.14 ± 6.39	14.53 ± 3.89	–30.61 ± 5.03
Cyclooxygenase-2 complexes energy components (kcal/mol)							
C25	–27.77 ± 4.38	–2.08 ± 2.39	5.95 ± 2.73	–3.63 ± 0.3	–29.86 ± 5.68	2.32 ± 2.58	–27.54 ± 4.45
C27	–28.72 ± 3.18	–1.25 ± 2.27	7.64 ± 2.98	–3.77 ± 0.25	–29.98 ± 3.81	3.87 ± 2.88	–26.11 ± 3.75
C43	–32.44 ± 6.56	–3.24 ± 6.56	14.05 ± 5.91	–4.53 ± 0.6	–35.68 ± 8.74	9.52 ± 5.51	–26.16 ± 5.49
Curcumin	–30.65 ± 7.53	–17.54 ± 10.23	36.45 ± 11.93	–4.33 ± 0.66	–48.2 ± 13.63	32.12 ± 11.58	–16.07 ± 7.97
Ibuprofen*	–33.66 ± 2.01	–7.45 ± 4.72	12.68 ± 2.71	–2.95 ± 0.1	–41.11 ± 4.95	9.73 ± 2.71	–31.38 ± 4.14

a Data presented as means ± SD.

b Are positive controls.

C25, C27, and C43 once again showed consistently good binding with Δ*G*_bind_ values clustered around −27 kcal·mol^–1^ (C25: −27.54 ± 4.45; C27: −26.11 ± 3.75; C43: −26.16 ± 5.49 kcal·mol^–1^). These energies are only slightly lower than the energies of the parent ligand ibuprofen (−31.38 ± 4.14 kcal·mol^–1^) in which indicating that the diarylpentanoids can stabilize the COX2 active site at a level comparable to a clinically used NSAID. In comparison, the free energy of binding of curcumin to COX2 is by far not as favorable and more variable (−16.07 ± 7.97 kcal·mol^–1^), primarily due to a large polar solvation penalty (EPB ≈ +36.45 kcal·mol^–1^) that offsets its stronger gas-phase electrostatic term. In the case of the derivatives, there is a stronger balance between the strong vdW contribution (∼−28 to −32 kcal·mol^–1^) and a more moderate PB penalty, producing tighter and more stable binding compared to curcumin in COX2.

Comparing the isoforms, all three derivatives hold constant or better Δ*G*_bind_ values in COX2 relative to their COX1 complexes compared to curcumin, which exhibits a reduction in affinity during the transition from COX1 to COX2 (from −27.19 to −16.07 kcal·mol^–1^). Combined with the MD data, these trends show that the diarylpentanoid scaffold, in particular C25 and C27, which attains a better and more dynamically stable balance of hydrophobic packing and solvation in COX2 compared to curcumin. Thus, this explains an energetic rationale for the stronger COX2-selective activity of these derivatives in the biochemical and cellular assay.

### 3.6 Effect of Curcumin Derivatives on COX1 and COX2 Gene Expression in IFN-γ/LPS-stimulated RAW264.7 Cells

To identify the effects of the curcumin derivatives on inflammatory signaling at the transcriptional level, the expression of COX1 and COX2 mRNA under the influence of compounds 25, 27, and 43 in IFN-γ/LPS- and PMA-stimulated RAW264.7 macrophages was analyzed. The stimulation of cells with IFN-γ/LPS and PMA significantly increased transcripts of COX2 and COX1, respectively (Figure 7).

**Figure 7:**
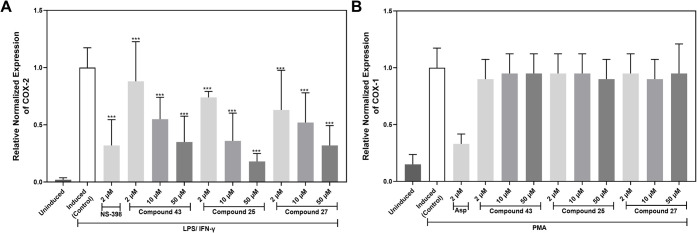
Effects of compounds 25, 27, and 43 on COX1 and COX2 gene expression in stimulated RAW264.7 cells. (A) Cells (2 × 10^5^ cells/well) were incubated with recombinant murine IFN-γ (100 U/mL) and LPS (5 μg/mL) for 24 h, with or without the compounds. NS-398 (2 μM) represents the positive control for COX2. (B) Cells (2 × 10^5^ cells/well) were incubated with PMA (10 nM) for 24 h, with or without the compounds. Aspirin (2 μM) was used as a positive control for COX1 transcriptional modulation to avoid high-dose cellular stress effects while maintaining biological relevance. The total RNA was extracted, and gene expression was analyzed using RT-qPCR, normalized to GAPDH and β-actin levels. Data are presented as mean ± SEM from three independent experiments (*n* = 3). Statistical significance was determined using one-way ANOVA followed by Tukey’s post hoc test. ***P < 0.001 indicates a significant difference compared to the respective stimulated control group.

All of the tested compounds reduced the expression of COX2 in a dose-dependent manner. C25 at its highest concentration (50 μM) provided the maximum inhibition with a reduction of COX2 mRNA by approximately 82%, while C27 and 43 reduced it by up to 68.4% and 64.6%, respectively. Lower concentrations (10 and 2 μM of the tested compounds) still significantly reduced COX2 transcripts, meaning that their suppressive actions run to lower micromolar levels (Figure 7b). These trends are highly comparable to the potency observed in the PGE_2_ inhibition assay, and this demonstrates that transcriptional downregulation of COX2 is also one of the major contributing mechanisms.

In comparison, interestingly, the COX1 expression was not affected by the compounds at all concentrations being tested (2–50 μM). It respectfully failed to provide a significant variation in response compared with the optimized control and did not portray a dose response curve. This bias in the inhibition of COX2 transcription, unlike intact COX1 expression, is a major anti-inflammatory advantage over classical nonselective NSAIDs. The observed selectivity can be subjected to the COX enzymatic inhibition profiles, where compounds 25 and 43 selectively inhibited COX2. All of these data show that the curcumin derivatives and compound 25 selectively inhibit the transcription of COX2 without affecting the expression of basal COX1. This molecular selectivity justifies their future as effective COX2-targeted anti-inflammatory candidates and provides mechanistic support for their phenotypic processing of the PGE_2_ production.

## 4 Discussion

This present study provides an integrated experimental–computational framework for describing how the structural alterations of curcumin-derived diarylpentanoids can be used to control the synthesis of PGE_2_ through selective modulation of the COX pathway. Of all 43 derivatives, three compounds, C25, C27, and C43, were observed to be potential modulators of inflammatory signaling, with C25 and C43 demonstrating the stronger potency, COX2 selectivity, and molecular stability. The result of the biochemical assays, docking profiles, long-time scale molecular dynamics, and MM/PBSA energetics is used to collectively develop a consistent mechanistic foundation of COX2-selective inhibition to further our understanding of diarylpentanoid anti-inflammatory scaffolds.^24^

The pronounced activity of cyclohexanone-linked derivatives, especially C25 and C27, indicates the significance of the rigidity of the linker in the stabilization of pharmacophoric geometry. In line with previous crystallographic and computational studies, six-membered cyclohexanone rings closely mimic the β-diketone enol conformation of curcumin, which provides better steric complementarity with the COX catalytic channel. *Ortho*- or *para*-fluorine atoms found in C25 and C27 are electron-withdrawing groups that further increase the activity by increasing the polarization of the aryl rings and reinforcing hydrophobic contacts in the COX pocket. These observations are supported by QSAR modeling, which found lipophilicity (ALogP) and molecular flexibility (NumRotatableBonds) as positive predictors of the activity and the negative effect of Kappa_2 indicates that overbranched or highly complex scaffolds are counterproductive to binding activity. Therefore, from these structure–activity trends, it is concluded that optimized PGE_2_ inhibition is initiated by the derivatives with balanced rigidity, hydrophobic surface area, and appropriately positioned electron-withdrawing groups.

The consistent suppression of PGE_2_ by active derivatives prompted investigation into isoform-specific COX inhibition; however, many compounds showed preference toward COX1, with only C25 and C43 showing significant COX2 selectivity, a pharmacological advantage over other NSAIDs, which usually lead to gastrointestinal toxicity because of unwanted COX1 inhibition.

Docking studies have shown that C25 and C43 occupy varied numbers of noncovalent interactions in COX2, which include engaging hydrophobic, π-stacking, as well as, in the instances of C25 and C43, halogen or hydrogen bonds that are absent in COX1. C27, despite carrying fluorine substitutions, lacks these stabilizing interactions and exhibits higher affinity for COX1, underscoring the importance of interaction geometry rather than substituent presence alone.

Molecular dynamics simulations provide a more detailed understanding of the effect of the derivatives on the structural integrity of COX isoforms at physiologically relevant time scales. From the 500 ns simulations, all tested compounds consistently induced lower RMSD values and stable residue mobility (RMSF) in the COX2 catalytic channel in comparison to curcumin. From the stable *R*_g_ profiles, these derivatives have been shown to maintain tighter compactness, thus suggesting a strong stabilizing influence on the COX2 architecture. Comparatively, fluctuating patterns of the classical NSAIDs indicate disparities in the ligand-mediated plasticity and demonstrate the potential of diarylpentanoids to be more conformationally stabilizing inhibitors. Surprisingly, C25 and C43 that preferentially stabilize COX2 through both enthalpic and entropic contributions add to their high biological effectiveness, indicating that the alignment between dynamic behavior and enzymatic inhibition strongly reinforces the mechanistic interpretation.^26^

The dynamic observations are further validated using MM/PBSA calculations by quantifying binding energetics.^27^ In the case of both COX isoforms, van der Waals interactions were the primary stabilizing force, which is consistent with the hydrophobic nature of the COX binding channel and the high lipophilicity of the derivatives. In COX2, C25 exhibited the most preferred binding free energy, closely followed by C43, with curcumin having the least affinity and unstable energy components. The COX2 energetics are not limited to the hydrophobic attraction, where the polar solvation penalties were minimized in C25, suggesting that its scaffold is in the most optimal desolvation-binding equilibrium. This efficiency is what contributes to the fact that C25 is quite effective even at lower concentrations in PGE_2_ inhibition assays. The fact that binding energetics, docking interactions, transcriptional suppression, and biological activity almost coincide is a strong indication that C25 and C43 are mechanically strong lead compounds.

Besides direct enzymatic inhibition, C25, C27, and C43 had a strong effect on COX2 mRNA expression in stimulated macrophages, with C25 having the most significant dose effect. Maintaining the expression of COX1 points to selective inhibition of the inflammatory pathway, as opposed to total inhibition of prostaglandin biosynthesis. This dual mechanism of diarylpentanoids, direct catalytic inhibitor/transcriptional down-regulator, implies that diarylpentanoids could potentially produce a longer-term effect of attenuating COX2-mediated signaling than NSAIDs, which only attenuate by their enzymatic action. Their translational potential in next-generation anti-inflammatory agents is therefore improved by the convergence of the enzymatic and transcriptional effects.^26^

Together, the findings reveal that C25 and C43 are highly potential scaffolds, which is proven by their intrinsic COX2 selectivity, good PGE_2_ inhibition, and favorable dynamic and energetic properties. Their cyclohexanone linkers, strategic fluorination, and optimized hydrophobic profiles provide explicit design guidelines in the development of future analogues. However, there are several weaknesses that are worth noting. The current work focuses on in vitro macrophage models, which may not be able to fully capture in vivo pharmacokinetics, metabolic stability, or off-target signaling. Future research ought to test these derivatives in animal models of inflammation, determine their ADME properties, conduct site-directed mutagenesis to confirm the existence of predicted binding residues, and investigate broader structure–activity optimization.^28^ The combined biochemical measurements, gene expression profiling, structure–activity analysis, molecular docking, long-time scale MD simulations, and binding energy calculations pipeline create a consistent mechanistic picture of the high activity of C25 and C43. These discoveries promote the medicinal chemistry of curcumin-derived diarylpentanoids and their possible use as the next generation of anti-inflammatory agents by balancing selective COX2 inhibitors with mechanistic benefits over nonselective NSAIDs and curcumin itself.

## 5 Conclusion

This work has found curcumin-derived molecules that showed improved anti-inflammatory properties and COX2 selectivity. Of the 43 derivatives that were synthesized and tested in IFN-γ/LPS-stimulated RAW 264.7 macrophages, C25, C27, and C43 showed significant, dose-dependent inhibition of PGE_2_ production with significantly lower IC_50_ values than curcumin and no cytotoxicity. The analysis of QSAR showed that suppressing PGE_2_ is favored by electron-withdrawing substituents, patterns of aryl substitution, and increased lipophilicity. Computational studies also supported these results: molecular docking and long (500 ns) MD simulations revealed that C25 and C43 are favorably oriented in the COX2 active site and offer more structural stabilization to COX2 than COX1 due to hydrophobic, π-cation, and halogen interactions. Their binding affinities were also supported using *in silico* simulations (MM/PBSA calculations), and C25 had the most favorable energetic interaction profile coupled with a distinct preference toward COX2. Collectively, the biological and computational findings converge to provide C25 and C43 with good leads toward future therapeutic opportunities as promising COX2-selective anti-inflammatory agents.

## Supplementary Material



## Notes

Primer sequences for RT-qPCR, a list of descriptors calculated for the QSAR model, interacting amino acid residues at the COX binding sites identified via docking, and MM/PBSA decomposition data for each ligand used in the study (PDF)

## Abbreviations

COX1: Cyclooxygenase plays a role in gastric mucosal protection, platelet aggregation, and kidney renal activity
COX2: Cyclooxygenase reaction to inflammatory cues, including lipopolysaccharide LPS, interleukin-1β, tumor necrosis factor-α, and interferon-γ
PGE_2_: Prostaglandin E2
NSAIDs: Nonsteroidal anti-inflammatory drugs
IFN: Interferon-gamma
IC_50_: Half maximal inhibitory concentration
QSAR: Quantitative Structure–Activity Relationship
MD: Molecular Dynamics
LPS: Lipopolysaccharide
NF-kB: Nuclear factor kappa-light-chain-enhancer of activated B cells
MM/PBSA: Molecular Mechanics/Poisson–Boltzmann Surface Area
RMSD: Root-mean-square deviation
RMSF: Root-mean-square fluctuation
CHARMM-GUI: Chemistry at HARvard Macromolecular Mechanics-Graphical User Interface
