# Supplementary material for: Integrated Experimental and Computational Profiling of Curcumin-Derived Diarylpentanoids Reveals Mechanistic Determinants of COX1/COX2 Inhibition and Selectivity
Source: ACS Omega. 2026 Jun 3;11(23):33642–54. doi: 10.1021/acsomega.5c13194 (PMC13280910; doi:10.1021/acsomega.5c13194)
Supplement: Supplementary file 1 [file ao5c13194_si_001.pdf]

Integrated Experimental and Computational Profiling of Curcumin-Derived Diarylpentanoids Reveals Mechanistic Determinants of COX1/COX2 Inhibition and Selectivity

Mohammad Nazri Abdul Bahari[a], Nurul Hana Mas'od[b], Kamal Rullah[c], Mohd Fadhlizil Fasihi Mohd Aluwi[d], Kok Wai Lam[e], Faridah Abas[f], Wan Mardhiyana Wan Ayub[b], Muhamad Arif Mohamad Jamali[b], Asrulnizam Abd Manaf[a*], Syahida Ahmad[g*]*

[a]Collaborative Microelectronic Design Excellence Center, Universiti Sains Malaysia, Persiaran Bukit Jambul, 11900 Bayan Lepas, Pulau Pinang, Malaysia

[b]Faculty of Science and Technology, Universiti Sains Islam Malaysia, 71800 Nilai, Negeri Sembilan Darul Khusus, Malaysia

[c]Kulliyyah of Pharmacy, International Islamic University Malaysia Kuantan Campus, Jalan Sultan Ahmad Shah, Bandar Indera Mahkota, 25200 Kuantan, Pahang Darul Makmur, Malaysia

[d]Faculty of Industrial Sciences and Technology, Universiti Malaysia Pahang Al-Sultan Abdullah, 26600 Kuantan, Pahang, Malaysia

[e]Centre for Drug and Herbal Development, Faculty of Pharmacy, Universiti Kebangsaan Malaysia, Jalan Raja Muda Abdul Aziz, 50300 Kuala Lumpur, Malaysia

[f]Natural Medicines and Products Research Laboratory, Institute of Bioscience, Universiti Putra Malaysia, 43400 UPM Serdang, Selangor, Malaysia

[g]Faculty of Biotechnology and Biomolecular Sciences, Universiti Putra Malaysia, 43400 UPM Serdang, Selangor, Malaysia

**Corresponding Authors*

Supplementary Information

Table S1 Primers sequences for *COX-1*, *COX-2*, *ß-actin* and *GAPDH* used in RT-qPCR.

Gene	Primer sequences	Accession number	Sources
COX-1	Forward: TGGTGGATGCCTTCTCTCG<br>Reverse: AACAGATGGGATTCCCTAGGA	BC005573	Strausberg et al., 2002
COX-2	Forward: TGATCGAAGACTACGTGCAAC<br>Reverse: TCATCTCTCTGCTCTGGTCAA	NM_011198.5	Chen et al., 2024
GAPDH	Forward: ACCGCATCTTCTTGTGCAGT<br>Reverse: GCCAAAGTTGTCATGGATGA	AF106860.2	Zheng & Ramirez, 2000
ß-actin	Forward: TGGAATCCTGTGGCATCCATGAAAC<br>Reverse: TAAAACGCAGCTCAGTAACAGTCCG	NM_007393	Uchino et al., 2024

COX-1: cyclooxygenase 1, COX-2: cyclooxygenase 2, GAPDH: Glyceraldehyde 3-phosphate dehydrogenase, ß-actin: Beta-actin

Table S2 List of descriptors calculated for the models:

Descriptor class	Symbol	Description
Lipophilicity Descriptor	ALogP	Log of the octanol-water partition coefficient using Ghose and Crippen's method (Ghose and Crippen, 1986).
Molecular Property Counts	Num_RotatableBonds	Defined as single bonds between heavy atoms that are both not in a ring and not terminal.
Topological descriptor	Kappa_2	Kappa Shape Indices

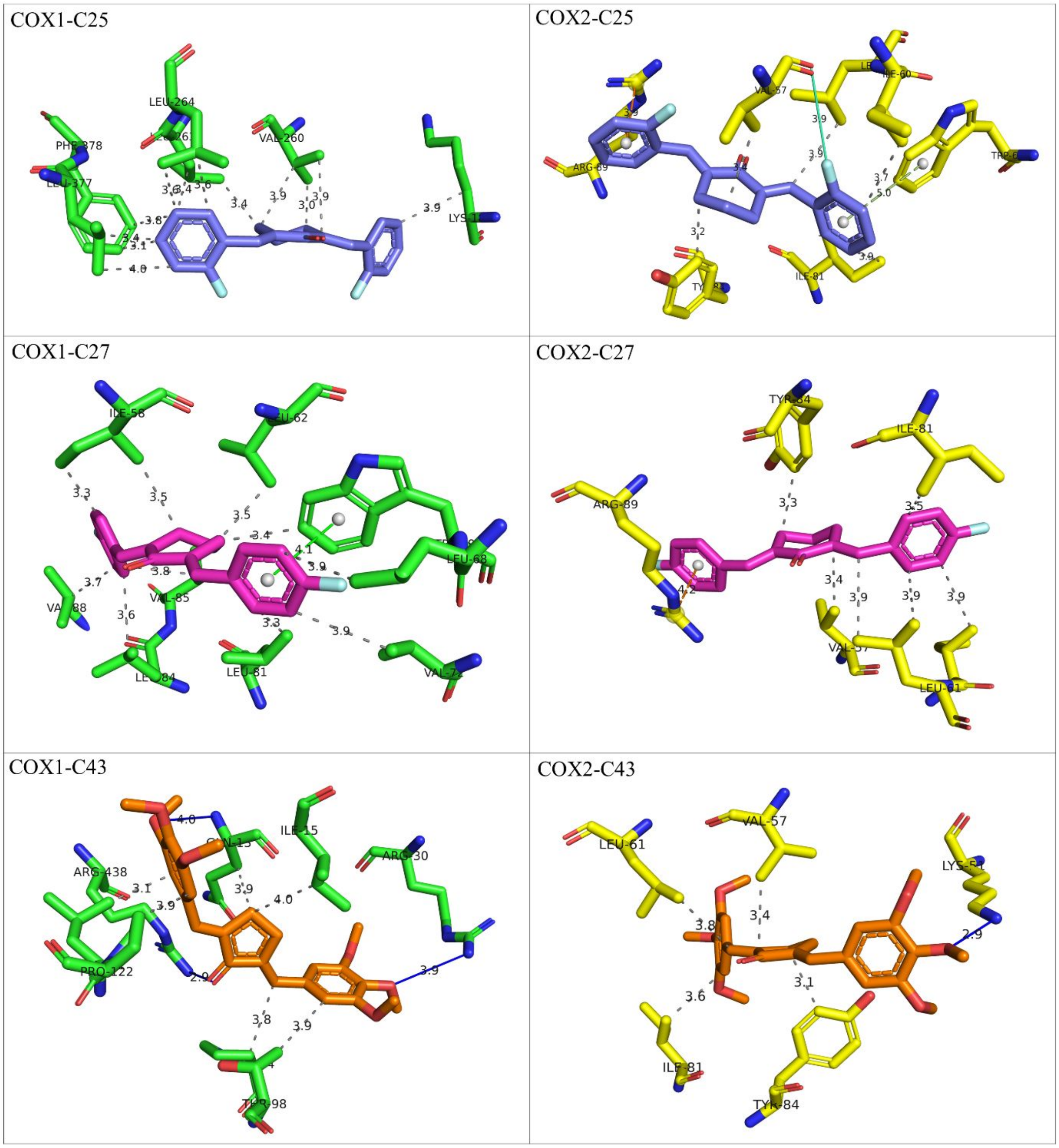

Figure S1. Interacting amino acid residues at the binding site of each and their distances in Angstrom (Å) with ligand towards the receptor (COX1 and COX2).

Table S3a Distances (Å) between ligands and amino acid residues of COX-1, categorized by interaction type (hydrophobic, π-stacking, hydrogen bond) and total number of interactions per ligand.

Distance between ligand and amino acid residues (Å)																
Types of Interaction	Hydrophobic Interaction					Pi-stacking					H-bond					Total Number of Interactions
Residues/Samples	C25	C27	C43	Cur	MXM	C25	C27	C43	Cur	MXM	C25	C27	C43	Cur	MXM	
CYS10														3.39		1
GLN13			3.91										3.32		2.0	3
ILE15			3.99													1
THR29														1.94		1
ARG30													2.98			2
ARG48															2.17	1
ARG52															2.95	1
ILE58		3.26														1
LEU62		3.54														1
LEU68		3.87														1
TRP69		3.38					4.07									2
VAL72		3.9														1
LEU81		3.31														1
LEU84		3.6														1
VAL85		3.78														1
VAL88		3.71														1
LEU92					3.68											1
PRO94			3.76													1
THR98			3.94											2.55		2
TYR99														1.87		1
LEU121			3.12	3.27												2
PRO122			3.92													1
LYS180	3.91															1
VAL260	2.97															1
LEU263	3.43															1
LEU264	3.57															1

LEU377	3.37															1
PHE378	3.05															1
GLU434														2.64		1
ARG438													1.96		3.02	1
GLY440															3.61	1
LYS442					3.63											1
Total Number of Interactions	6	9	6	1	2	-	1	-	-	-	-	-	3	5	5	38

Table S3b Distances (Å) between ligands and amino acid residues of COX2, categorized by interaction types (hydrophobic, π-stacking, π-cation, hydrogen bond, halogen bond) and total number of interactions per ligand.

Distance between ligand and amino acid residues (Å)																										
Types of Interaction	Hydrophobic Interaction					Pi-stacking					Pi-cation					H-bond					Halogen bond					Total Number of Interactions
Residues /Samples	C25	C27	C43	Cur	Ibu	C25	C27	C43	Cur	Ibu	C25	C27	C43	Cur	Ibu	C25	C27	C43	Cur	Ibu	C25	C27	C43	Cur	Ibu	
ARG12				3.49																						1
THR30				3.85																						1
VAL57	3.42	3.43	3.42																		3.89					4
ILE60	3.68	3.86																								2
LEU61	3.93	3.87	3.77																							3
TRP68						4.96																				1
ILE81	3.58	3.46	3.56																							3

TYR84	3.21	3.35	3.13															1.94								4
ARG89											3.87	4.16														2
TYR91									5.01										2.33							2
SER95																			1.99							1
VAL350					3.36																					1
TYR356					3.53																					1
LEU360					3.92																					1
TRP388					3.88																					1
ALA528					3.62																					1
Total No. of Interactions	5	5	4	2	5	1	-	-	1	-	1	1	-	-	-	-	-	1	2	-	1	-	-	-	-	29

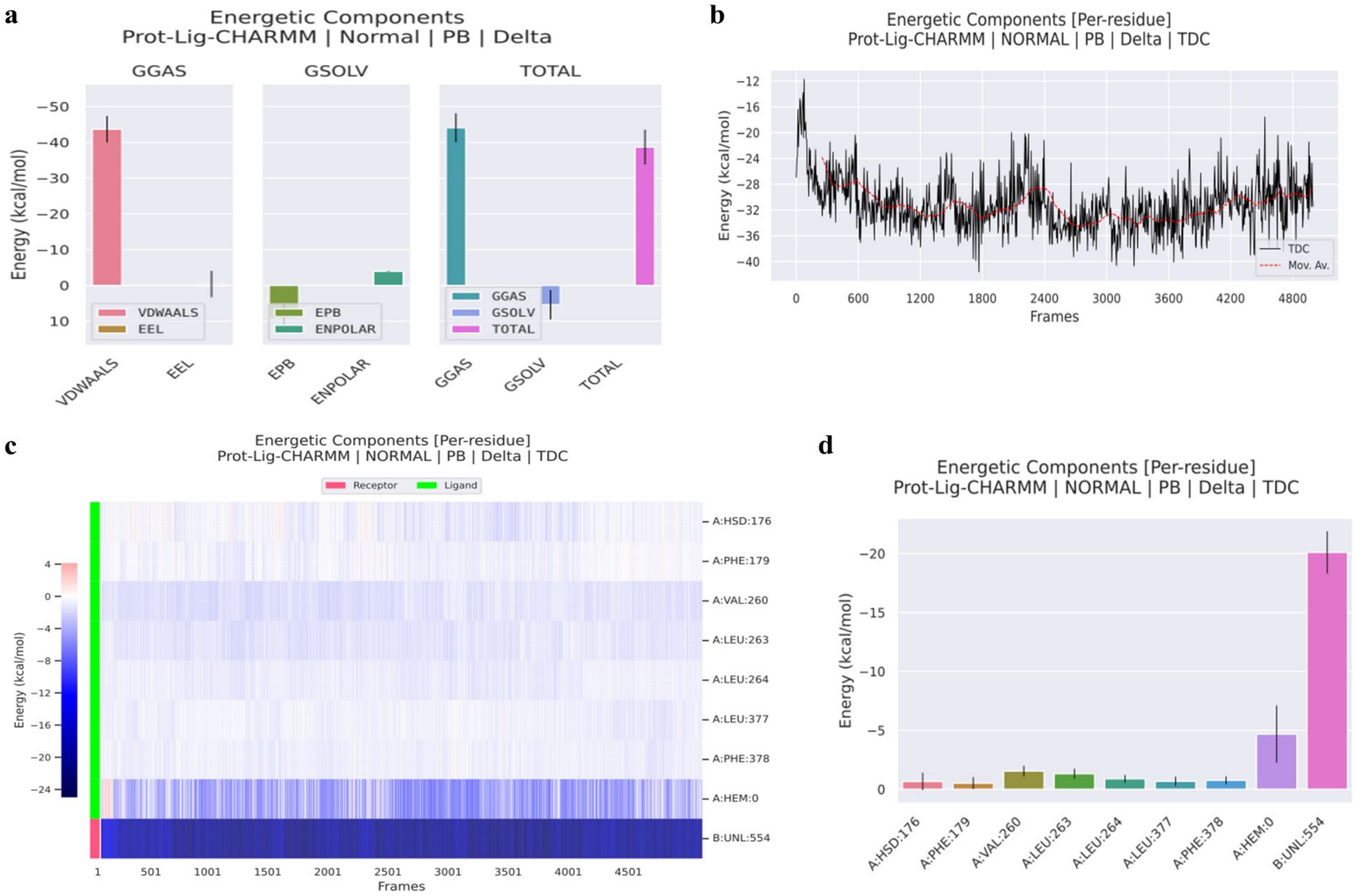

Figure S2 MM/PBSA Decomposition of COX1-C25 complex. (a) Energetic components; (b) Energetic components (Per residue); (c) Heatmap of energetic components (Per residue); (d) Histogram of energetic components (Per residue)

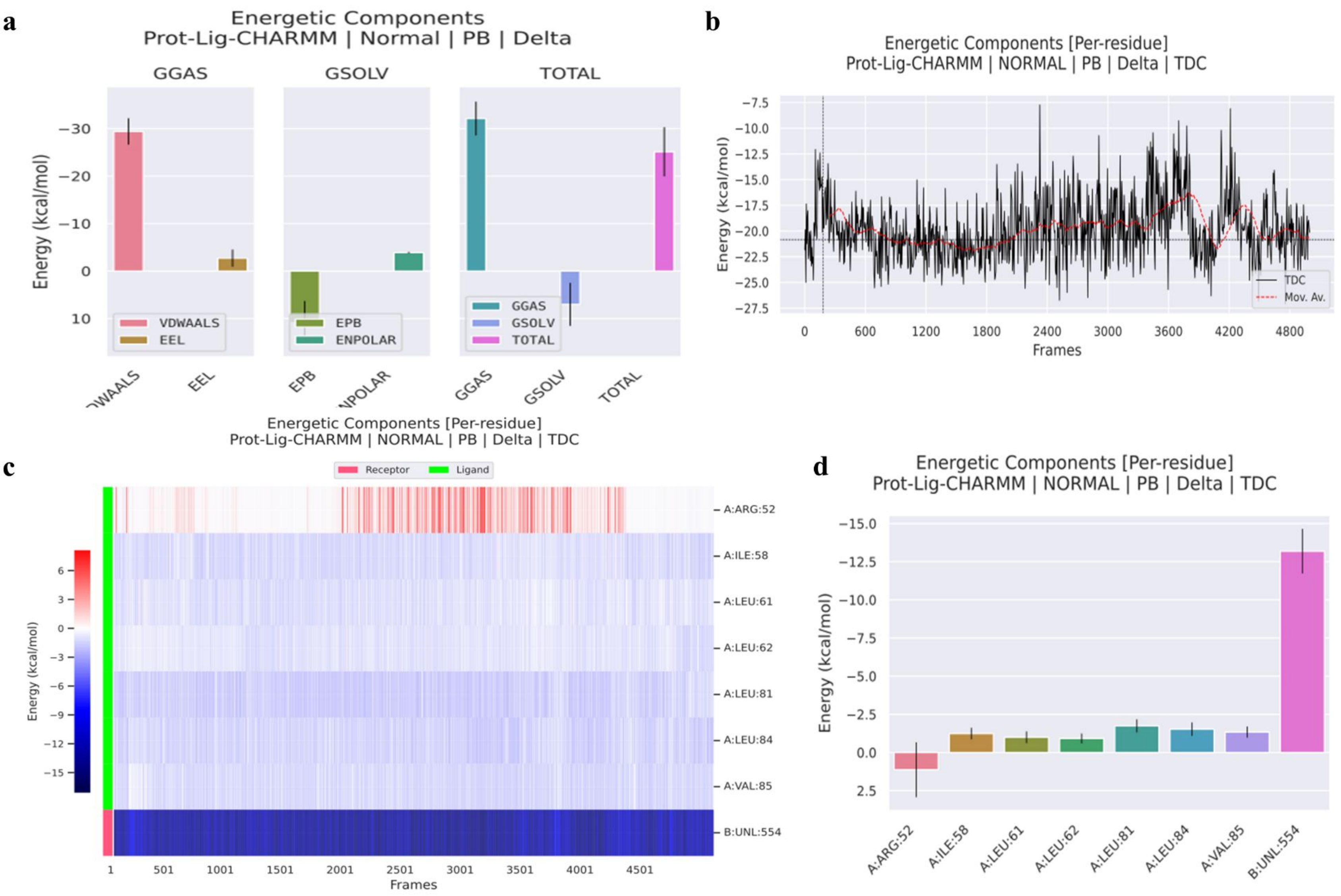

Figure S3 MM/PBSA Decomposition of COX1-C27 complex. (a) Energetic components; (b) Energetic components (Per residue); (c) Heatmap of energetic components (Per residue); (d) Histogram of energetic components (Per residue)

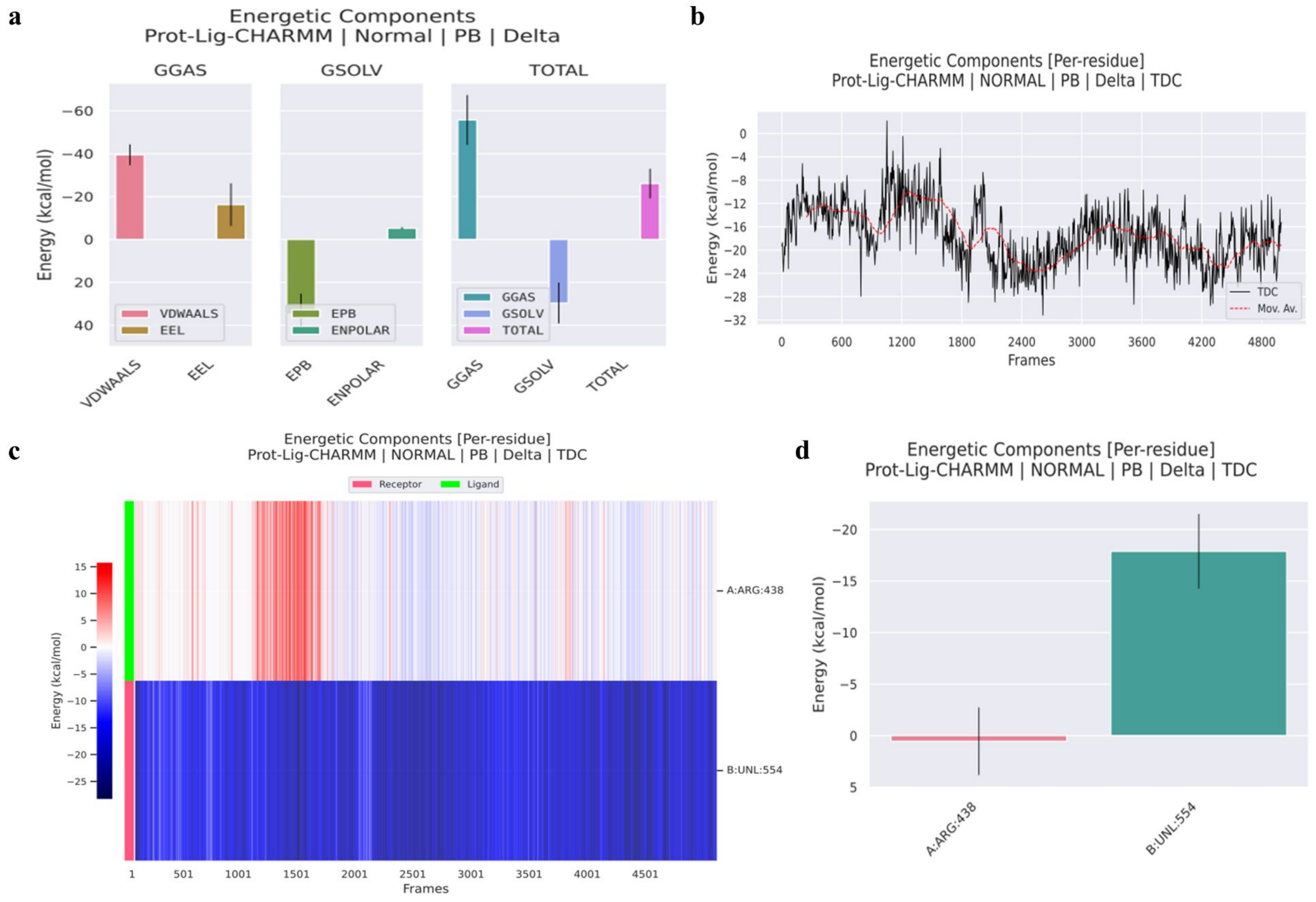

Figure S4 MM/PBSA Decomposition of COX1-C43 complex. (a) Energetic components; (b) Energetic components (Per residue); (c) Heatmap of energetic components (Per residue); (d) Histogram of energetic components (Per residue)

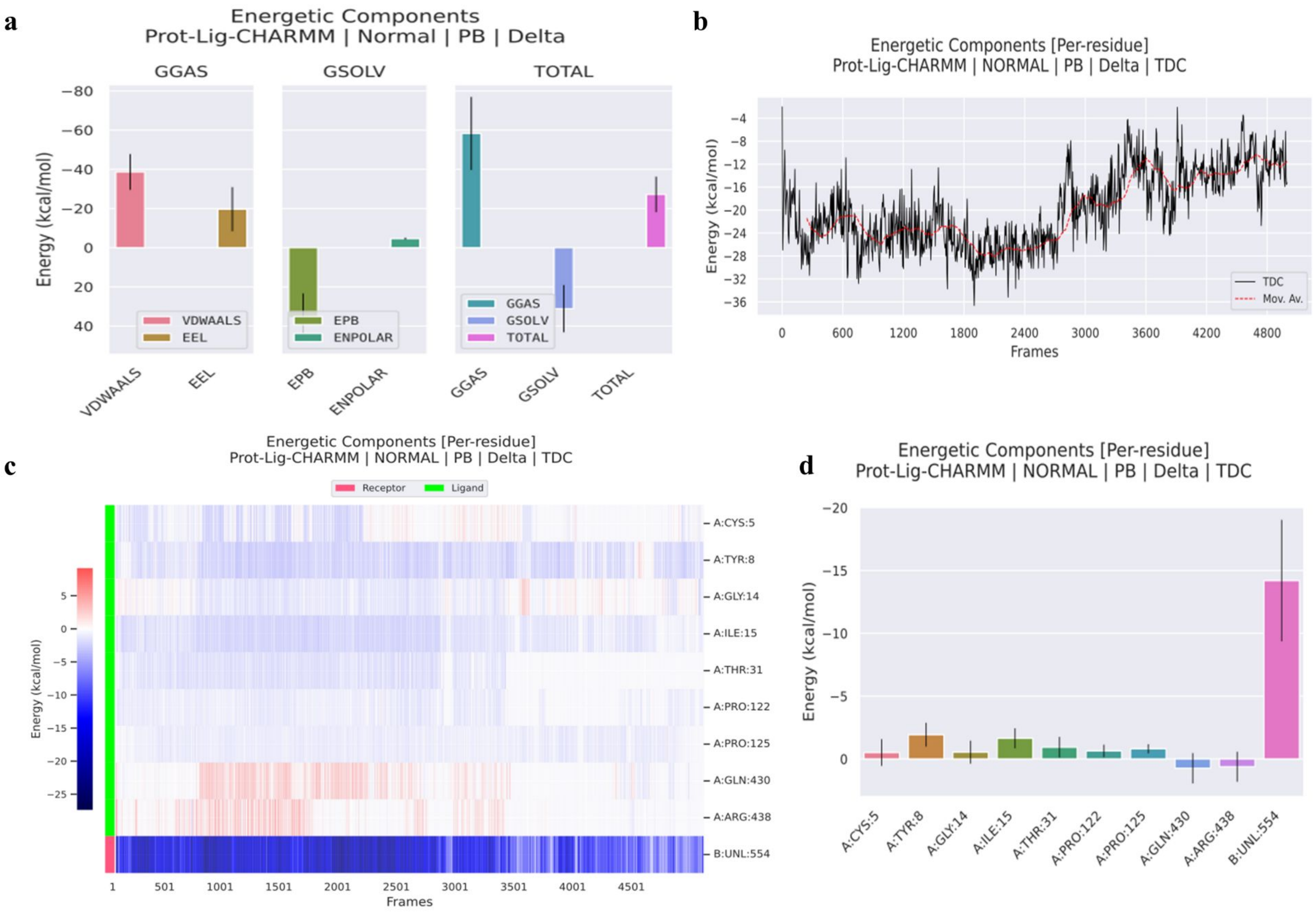

Figure S5 MM/PBSA Decomposition of COX1-Curcumin complex. (a) Energetic components; (b) Energetic components (Per residue); (c) Heatmap of energetic components (Per residue); (d) Histogram of energetic components (Per residue)

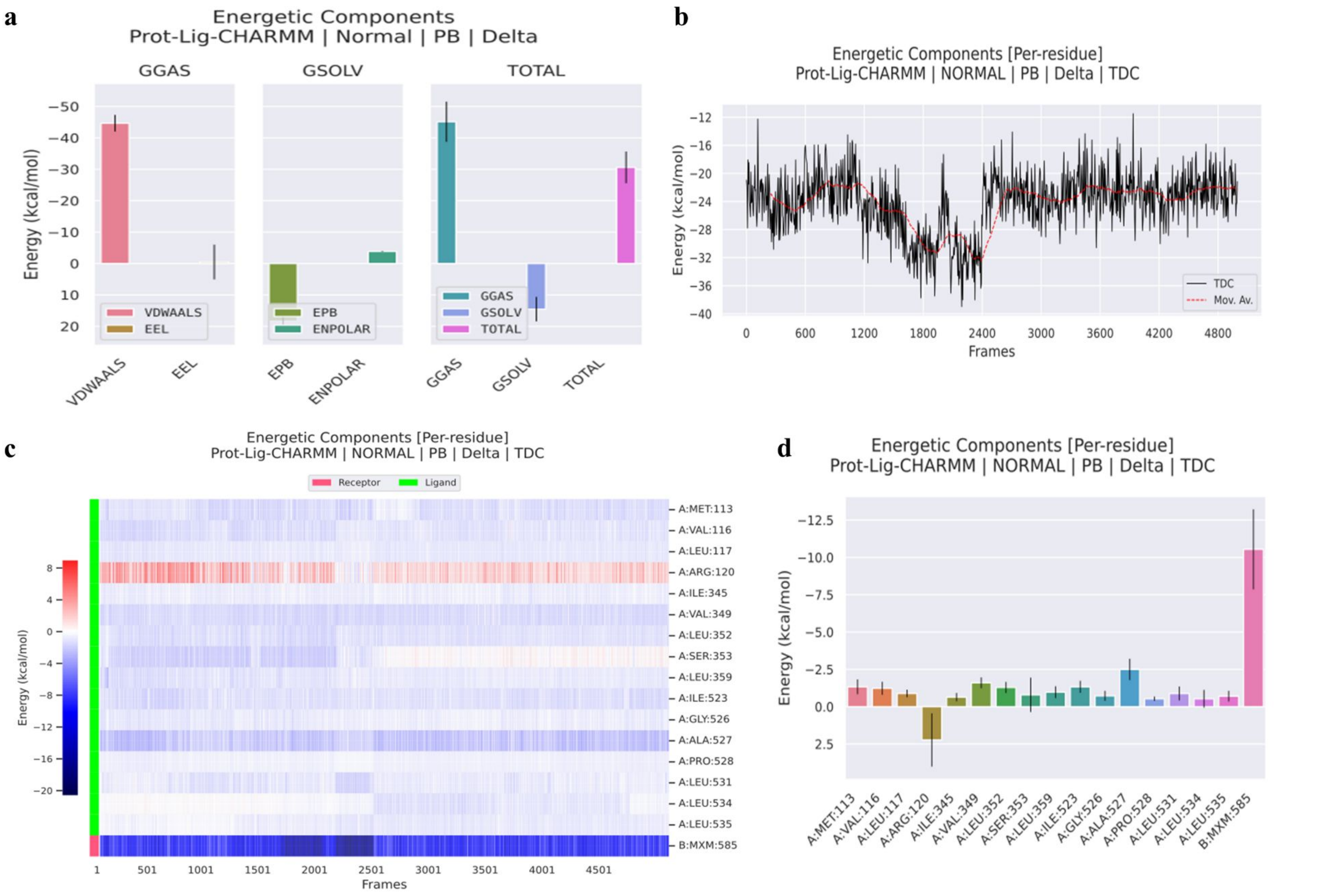

Figure S6 MM/PBSA Decomposition of COX1-MXM complex. (a) Energetic components; (b) Energetic components (Per residue); (c) Heatmap of energetic components (Per residue); (d) Histogram of energetic components (Per residue)

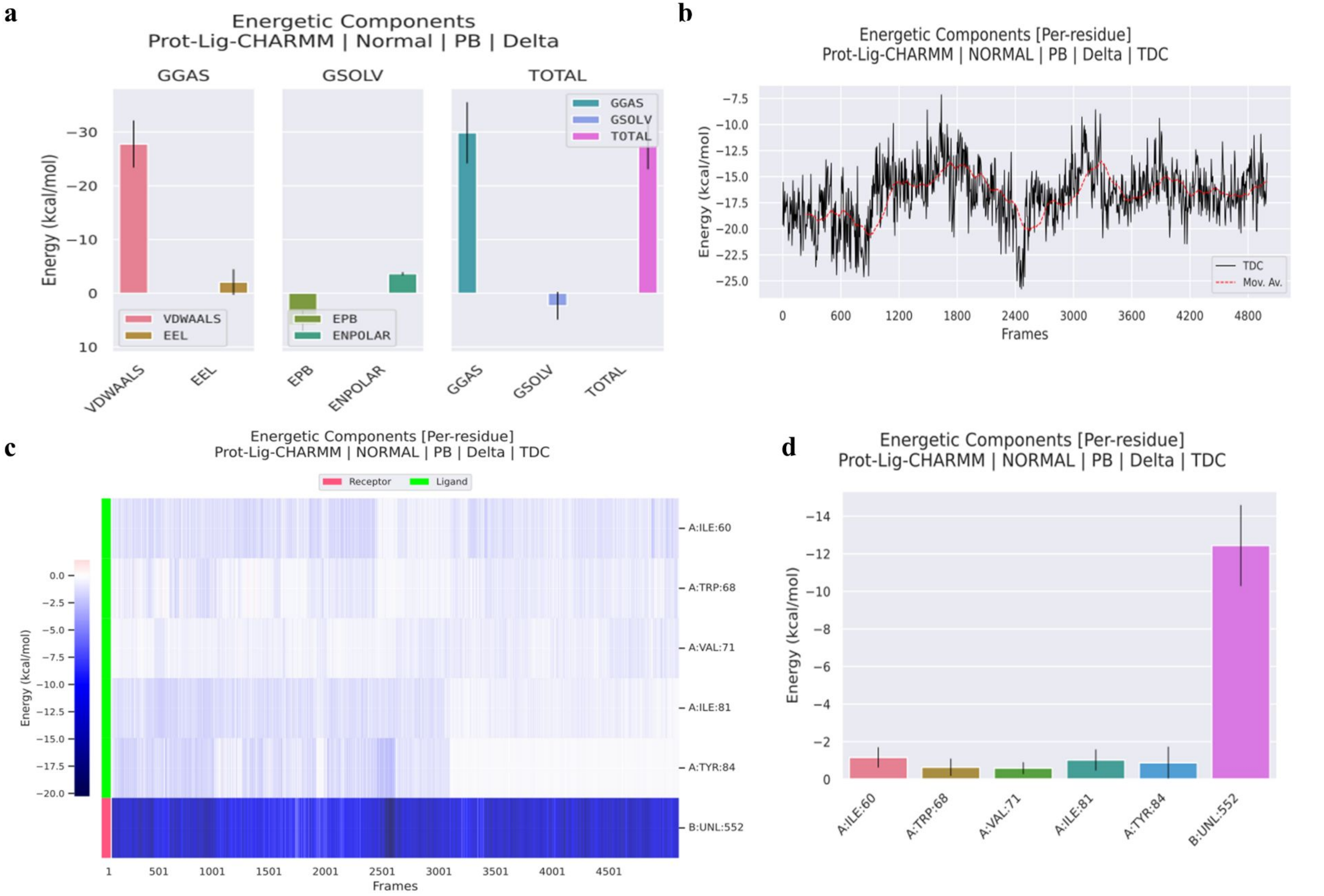

Figure S7 MM/PBSA Decomposition of COX2-C25 complex. (a) Energetic components; (b) Energetic components (Per residue); (c) Heatmap of energetic components (Per residue); (d) Histogram of energetic components (Per residue)

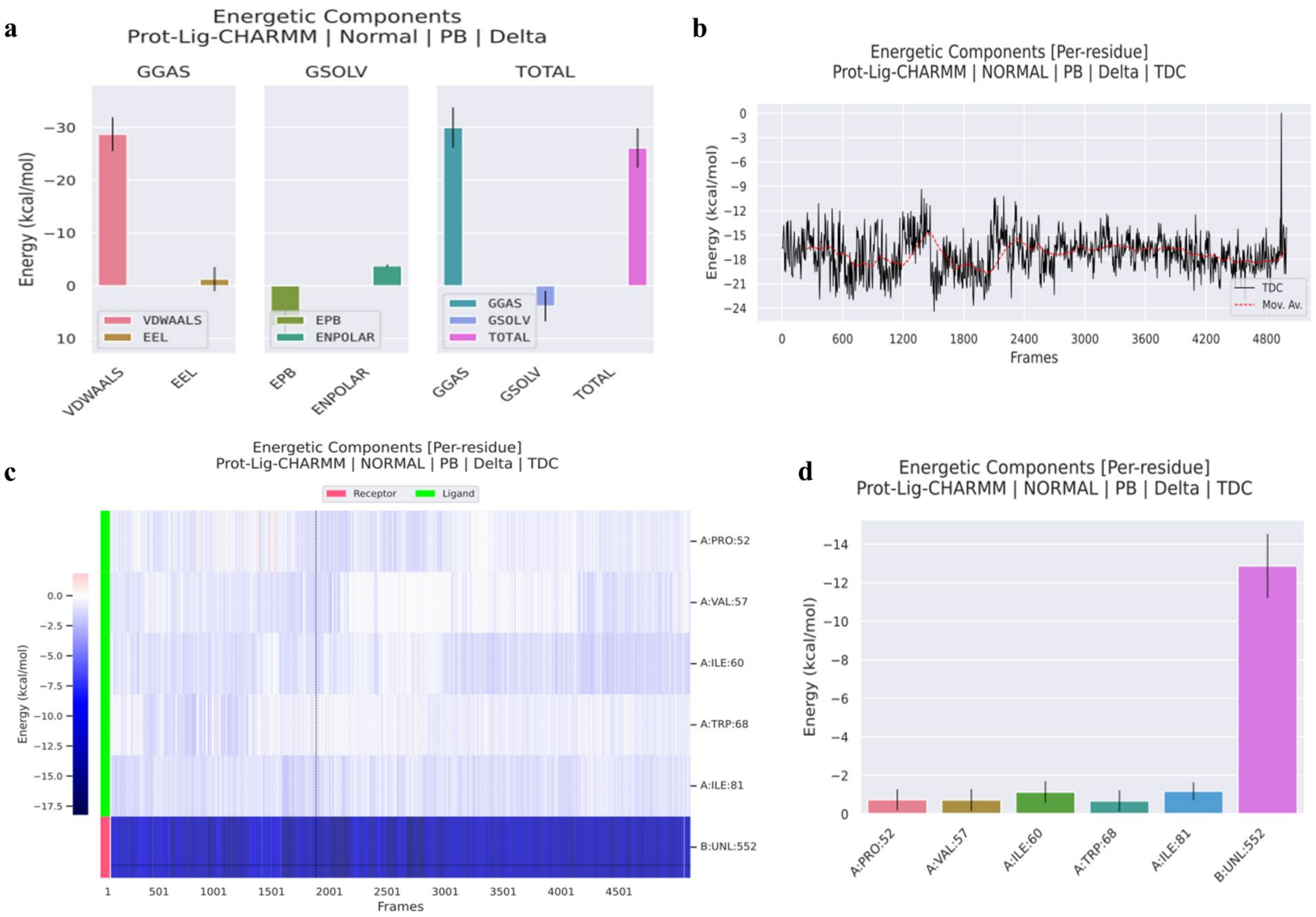

Figure S8 MM/PBSA Decomposition of COX2-C27 complex. (a) Energetic components; (b) Energetic components (Per residue); (c) Heatmap of energetic components (Per residue); (d) Histogram of energetic components (Per residue)

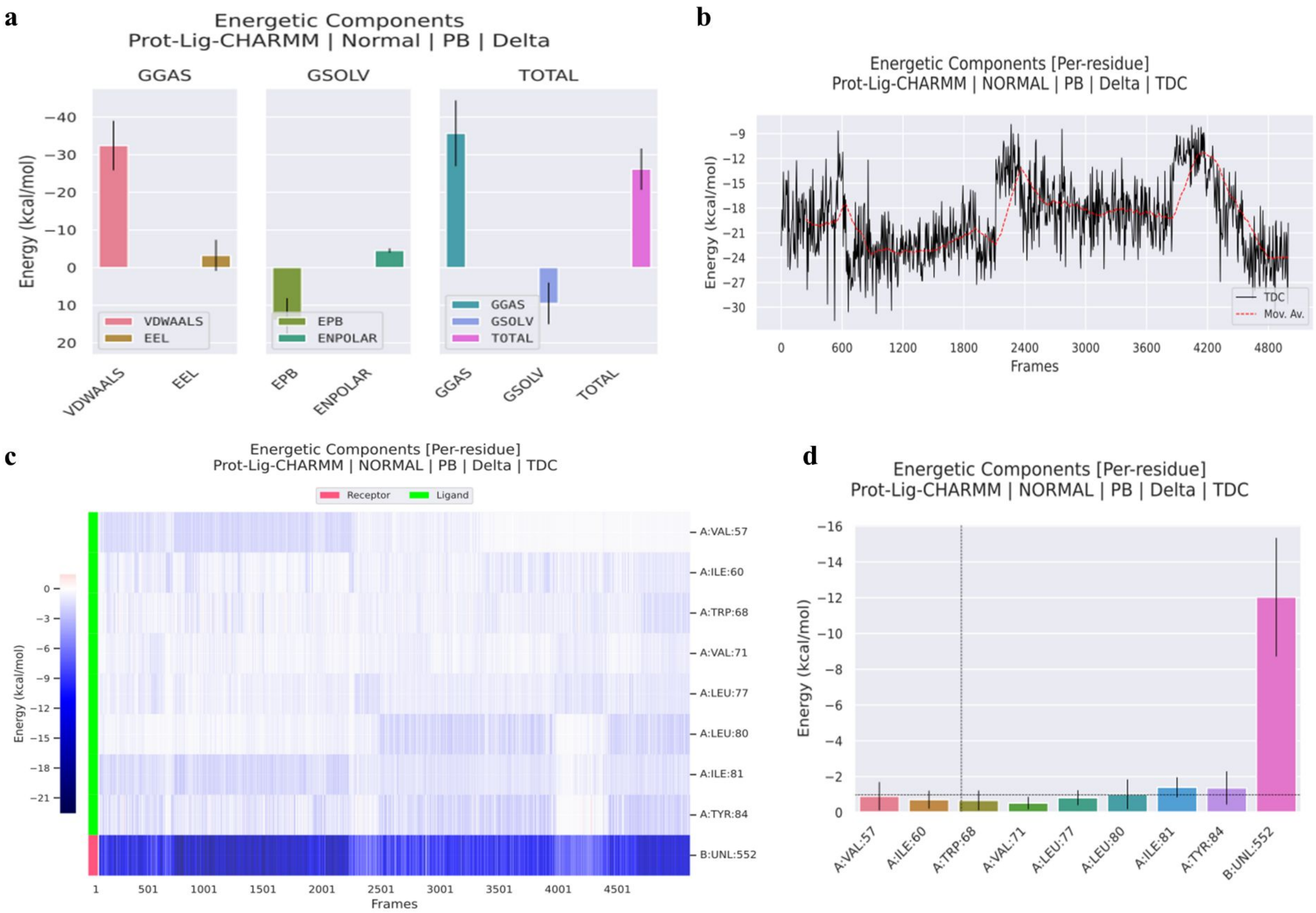

Figure S9 MM/PBSA Decomposition of COX2-C43 complex. (a) Energetic components; (b) Energetic components (Per residue); (c) Heatmap of energetic components (Per residue); (d) Histogram of energetic components (Per residue)

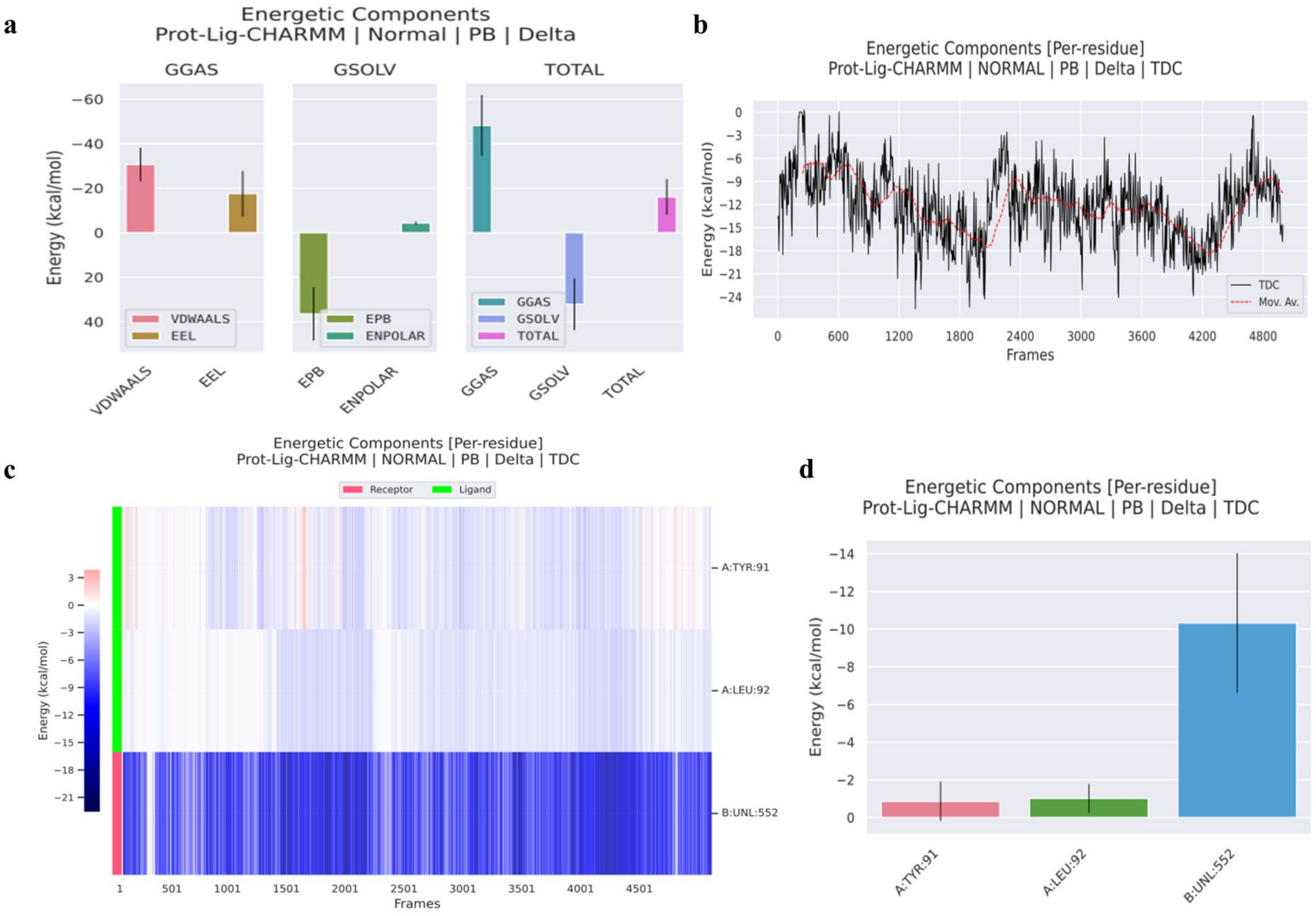

Figure S10 MM/PBSA Decomposition of COX2-Curcumin complex. (a) Energetic components; (b) Energetic components (Per residue); (c) Heatmap of energetic components (Per residue); (d) Histogram of energetic components (Per residue)

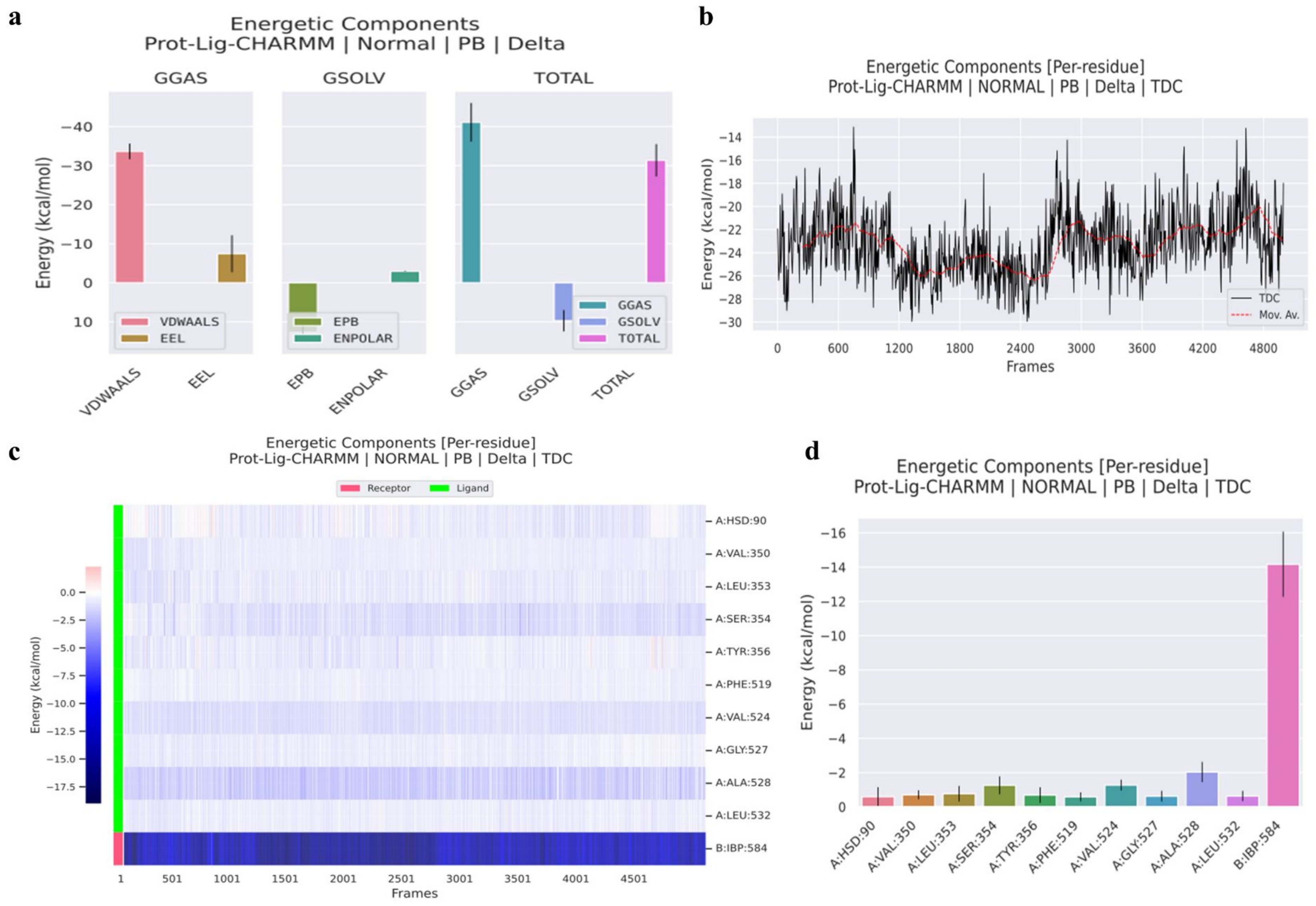

Figure S11 MM/PBSA Decomposition of COX2-Ibuprofen complex. (a) Energetic components; (b) Energetic components (Per residue); (c) Heatmap of energetic components (Per residue); (d) Histogram of energetic components (Per residue)

References:

1. Mammalian Gene Collection (MGC) Program Team*. Generation and initial analysis of more than 15,000 full-length human and mouse cDNA sequences. *Proceedings of the National Academy of Sciences* 2002, *99*(26), 16899-16903.
2. Chen, Y., Jian, X., Zhu, L., Yu, P., Yi, X., Cao, Q., ... & Li, J. PTGS2: A potential immune regulator and therapeutic target for chronic spontaneous urticaria. *Life Sciences* 2024, *344*, 122582.
3. Zheng, J., & Ramirez, V. D. Isolation of a glyceraldehyde-3-phosphate dehydrogenase (GAPDH) cDNA isoform from rat brain by a rapid PCR-based cloning method and its expression by RT-PCR. *National Center for Biotechnology Information* (NCBI) 2000, accession number AF106860.
4. Uchino, E., Kusumoto-Yoshida, I., Kashiwadani, H., Kanmura, Y., Matsunaga, A., & Kuwaki, T. Identification of hypothermia-inducing neurons in the preoptic area and activation of them by isoflurane anesthesia and central injection of adenosine. *The Journal of Physiological Sciences* 2024, *74*(1), 33.
